# Neurons in Primate Visual Cortex Alternate between Responses to Multiple Stimuli in Their Receptive Field

**DOI:** 10.3389/fncom.2016.00141

**Published:** 2016-12-27

**Authors:** Kang Li, Vladislav Kozyrev, Søren Kyllingsbæk, Stefan Treue, Susanne Ditlevsen, Claus Bundesen

**Affiliations:** ^1^Department of Mathematical Sciences, University of CopenhagenCopenhagen, Denmark; ^2^Department of Psychology, University of CopenhagenCopenhagen, Denmark; ^3^Cognitive Neuroscience Laboratory, German Primate CenterGoettingen, Germany; ^4^Bernstein Center for Computational NeuroscienceGoettingen, Germany; ^5^Chair Theory of Cognitive Systems, Institute for Neuroinformatics, Ruhr University BochumBochum, Germany; ^6^Visual Cognition Lab, Department of Medicine/Physiology, University of FribourgFribourg, Switzerland; ^7^Faculty for Biology and Psychology, Goettingen UniversityGeottingen, Germany

**Keywords:** probability-mixing, response-averaging, primate visual cortex, multiple stimuli, point process, model selection

## Abstract

A fundamental question concerning representation of the visual world in our brain is how a cortical cell responds when presented with more than a single stimulus. We find supportive evidence that most cells presented with a pair of stimuli respond predominantly to one stimulus at a time, rather than a weighted average response. Traditionally, the firing rate is assumed to be a weighted average of the firing rates to the individual stimuli (response-averaging model) (Bundesen et al., 2005). Here, we also evaluate a probability-mixing model (Bundesen et al., 2005), where neurons temporally multiplex the responses to the individual stimuli. This provides a mechanism by which the representational identity of multiple stimuli in complex visual scenes can be maintained despite the large receptive fields in higher extrastriate visual cortex in primates. We compare the two models through analysis of data from single cells in the middle temporal visual area (MT) of rhesus monkeys when presented with two separate stimuli inside their receptive field with attention directed to one of the two stimuli or outside the receptive field. The spike trains were modeled by stochastic point processes, including memory effects of past spikes and attentional effects, and statistical model selection between the two models was performed by information theoretic measures as well as the predictive accuracy of the models. As an auxiliary measure, we also tested for uni- or multimodality in interspike interval distributions, and performed a correlation analysis of simultaneously recorded pairs of neurons, to evaluate population behavior.

## 1. Introduction

The receptive field (RF) of a neuron in the visual system is the region within the visual field in which stimulation can affect the neuron's response. To understand visual information processing, it is fundamental to understand how the benefits of large RFs (integrating spatial information to allow encoding more complex and spatially extensive visual stimuli) are achieved without the loss of spatial precision caused by combining the responses to multiple stimuli in the RF into one response of the neuron.

In primary visual cortex, RFs are small, allowing for a direct high-resolution representation of stimulus position in retinotopic coordinates. Moving up the hierarchy of extrastriate visual areas, both in the temporal and dorsal pathways, RF sizes grow substantially (Smith et al., 2001; Gattass et al., 2005). This is generally seen as an adaptation to the functional specialization of these areas for more complex aspects of the visual environment, creating a need for integrating information over larger spatial areas, such as when encoding faces (Kanwisher and Yovel, 2006) in the ventral pathway or optic flow patterns (Gilmore et al., 2007) in the dorsal pathway. However, the benefit of spatial integration comes with the cost of a loss of information about the individual features when multiple stimuli fall in the RF, which happens frequently in mid- or high-level visual cortical areas (Orhan and Ma, 2015).

Most single-cell studies on processing in extrastriate visual cortex have focused on single stimuli, and most studies of responses to multiple stimuli have viewed the recorded activities as an integration of the responses that would have been evoked by each of the stimuli presented alone. This approach has led to the observation that the average firing rate to multiple stimuli is not the sum but rather a weighted average of the responses evoked by the individual stimuli when these are presented alone (Recanzone et al., 1997; Britten and Heuer, 1999; Reynolds et al., 1999; Zoccolan et al., 2005; Busse et al., 2009; Lee and Maunsell, 2009; MacEvoy et al., 2009; Reynolds and Heeger, 2009; Nandy et al., 2013). Here we show that looking only at the responses to multiple stimuli averaged across many trials has obscured the possibility that neurons multiplex the responses to the individual stimuli in time, shifting between response states dominated by individual stimuli (Bundesen et al., 2005; Bundesen and Habekost, 2008).

Reynolds et al. (1999) showed that a typical cell in visual area V2 or V4 responds to a pair of objects in its classical RF by adopting a rate of firing which, averaged across trials, equals a weighted average of the firing rates when objects are presented alone. We analyzed two opposing models, the two models being prototypes for how multiple stimuli are being processed on the single trial level, and both leading to the observed average behavior over trials. In the response-averaging model (e.g., Reynolds et al., 1999), the firing rate of a cell to a pair of stimulus objects in its classical RF is a weighted average of the firing rates to the individual objects. By contrast, in the probability-mixing model (Bundesen et al., 2005), the cell responds to the pair of objects as if only one of the objects were present in any given trial. Here we compare the abilities of the two models to account for spike trains recorded from single cells in area MT in response to (a) unidirectional moving random dot patterns (RDPs) presented singly in the RF and (b) nonoverlapping bidirectional pairs of such patterns in the RF. For unidirectional patterns, the two models coincide. Results from bidirectional pairs support the probability-mixing model over the response-averaging model.

## 2. Materials and methods

### 2.1. Experimental procedures

**Figure 1:**
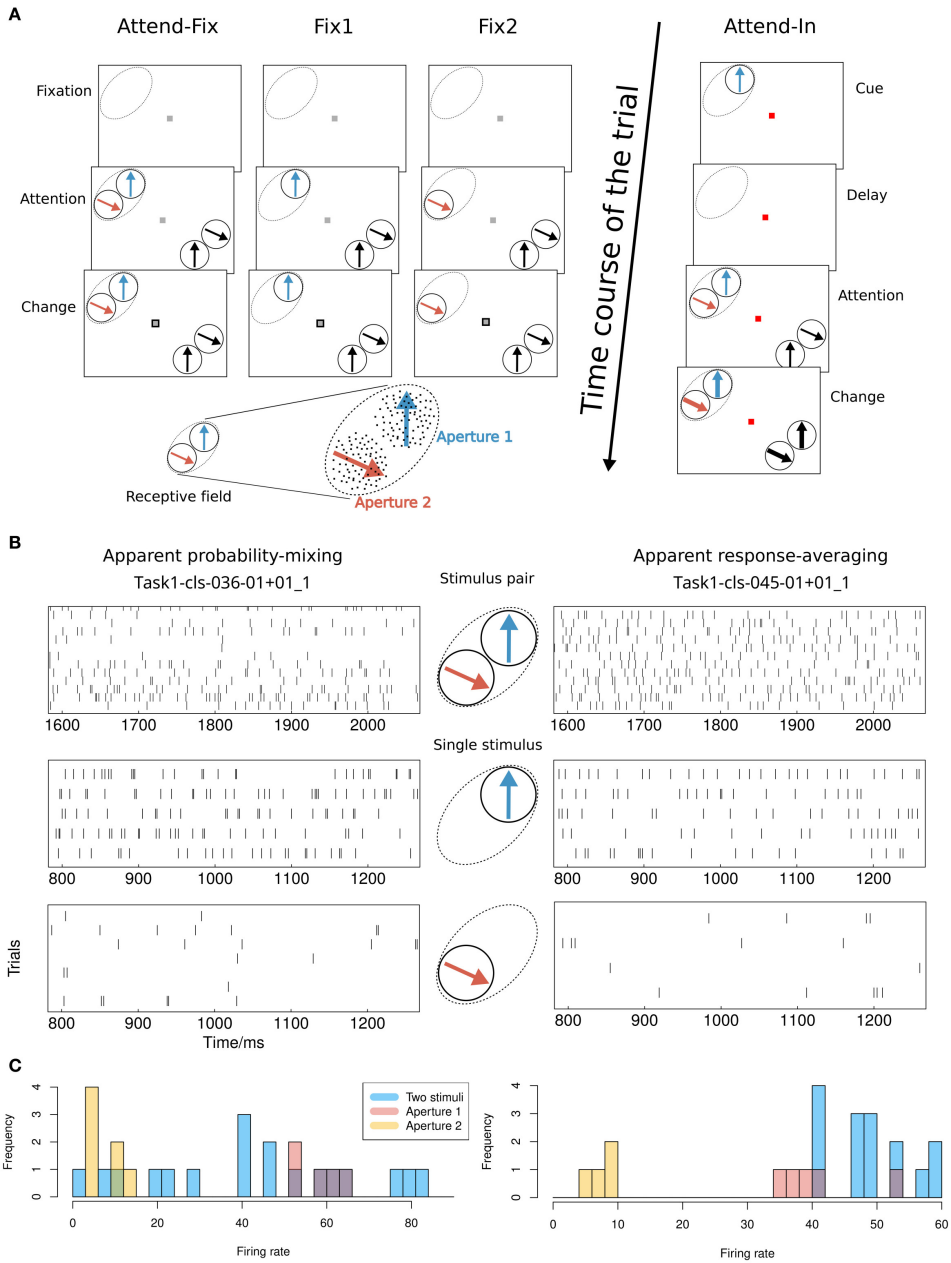
**Experimental setup and possible results**. **(A)** Visual stimuli and behavioral tasks. The lower left shows an example of the stimulus layout in the RF. The classical RF of an MT neuron is indicated by dashed ovals (not visible on the screen). Bidirectional motion patterns composed of two adjacent separated RDPs that moved within two stationary virtual apertures were used both inside and outside the RF. Apertures 1 and 2 were placed within the RF. In the bidirectional-motion condition *attend-in*, the monkey was required to detect a transient change in either speed or direction of motion of the cued target RDP. In the bidirectional-motion condition *attend-fix* and unidirectional motion conditions *fix1* and *fix2*, the monkey was required to detect a transient change in the luminance of the fixation spot. **(B)** Possible results. Illustration of the difference between the probability-mixing model and the response-averaging model by spike trains generated by stimulus pairs and single stimuli, respectively. The spike trains are taken from two neurons indicated in the scatter plots in **Figure 4A** as a square (apparent probability-mixing) and a triangle (apparent response-averaging). **(C)** Histograms of the empirical firing rates of the data in **(B)**.

The comparison between the response-averaging model and the probability-mixing model was performed by analysis of spike trains recorded from single cells in area MT. The data and computer code are available at Li et al. (2016). In this study, two rhesus monkeys were trained to perform visual tasks (see Figure 1A). Before each trial of the main experiment, a fixation spot (small red square) appeared in the middle of a computer screen. The monkey was trained to maintain its gaze on the fixation spot throughout each trial. It initiated a trial by pressing a lever. Immediately afterwards a cue was presented, which specified a target stimulus. The target, which could be either a RDP (*attend-in* condition) or the fixation spot (*attend-fix* condition), was later presented during the trial shown alone or together with distracting RDPs. The monkey was rewarded with a drop of juice for detecting a transient change in the target and responding by releasing the lever within 150–650 ms after the change.

In the *attend-fix* condition, the color of the fixation spot changed from red to gray when the monkey pressed the lever. The monkey was supposed to keep attention on the fixation spot. After 600 ms, two distractor RDPs were presented inside the RF of the recorded MT neuron and two were presented outside the RF (see Figure 1A). Each distractor pattern could change its motion (by increase in speed with 67% or clock- or counterclockwise change in direction by 45°) for a period of 130 ms beginning at a randomly chosen moment between 800 and 2400 ms after the onset of the RDPs. The monkey was required to detect a luminance change in the fixation spot which occurred within the same time window. For all cells, spike trains were recorded when two nonoverlapping patterns were simultaneously present in their RFs. For the majority of cells, spike trains were also recorded when only one pattern was present in the RF (at one of two locations, aperture 1 or 2, used for the bidirectional stimulation; see the *unidirectional conditions fix1* and *fix2* in Figure 1A).

In the *attend-in* condition, the fixation spot remained red during the whole trial. The cue was a moving RDP in aperture 1 presented for 600 ms. It had the same location and moved in the same direction as the target RDP. After the cue, a blank screen was shown for 800 ms (delay) followed by a display of the target RDP accompanied by three distractor RDPs. The first change in motion within the trial took place between 400 and 1200 ms after the onset of the patterns and could occur in either the target or one of the distractors. The transient change in speed or direction of motion was the same as the change used in the *attend-fix* condition. The target change took place inside aperture 1 in the RF of the recorded neuron.

**Figure 2:**
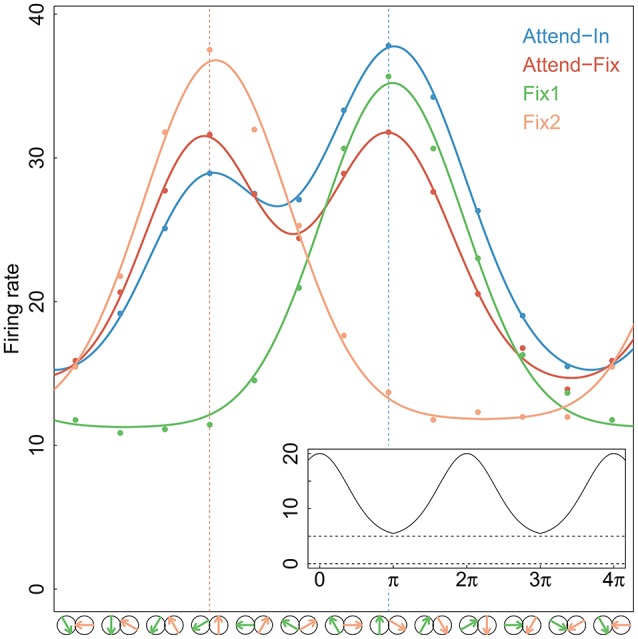
**Tuning curves**. Gaussian tuning curves (full-drawn lines) fitted to the average firing rates (dots) at each direction of movement and for each experimental condition of the 84 neurons that were tested in both bi- and uni-directional trials. In the analysis, each neuron has its own tuning curve, here an average curve is shown for illustration. The directions of movements in apertures 1 and/or 2 are shown by green and yellow arrows, respectively, along the *x*-axis. The two vertical dotted lines indicate the stimulus directions that were closest to the preferred direction, in aperture 1 (right) and aperture 2 (left). An example of the periodic Gaussian function, Equation (7), is shown in the insert on bottom-right, with parameters *A* = 15, *D* = 0, σ = 1.2, *r*_0_ = 5.

In the bidirectional conditions, direction of motion in aperture 2 was always 120° clockwise relative to that in aperture 1 (see Figure 1A). To determine a direction tuning curve for a neuron in a given condition (see Figure 2), both motion components were varied in steps of 30°. In all cells, full tuning curves were determined for the *attend-fix* and *attend-in* conditions. Recording of responses to the unidirectional components of the bidirectional stimuli, when each of the components was presented alone, provided two additional tuning curves.

#### 2.1.1. Monkey training and surgery

Two male rhesus monkeys (*Macaca mulatta*) were extensively trained to perform visual attentional tasks. The animals were implanted with a custom-made titanium implant to prevent head movements during training and recording, and a recording chamber (Crist Instruments, Hagerstown, MD, USA) on top of a craniotomy over the left (monkey C) or the right (monkey H) parietal lobe. The chamber positions were based on anatomical MRI scans.

All animal procedures of this study have been approved by the responsible regional government office [Niedersächsisches Landesamt für Verbraucherschutz und Lebensmittelsicherheit (LAVES)] under the permit numbers 33.42502/08-07.02 and 33.14.42502-04-064/07. The animals were group-housed with other macaque monkeys in facilities of the German Primate Center in Goettingen, Germany in accordance with all applicable German and European regulations. The facility provides the animals with an enriched environment (including a multitude of toys and wooden structures; Calapai et al., 2016), natural as well as artificial light, exceeding the size requirements of the European regulations, including access to outdoor space. Surgeries were performed aseptically under isoflurane anesthesia using standard techniques (see Martinez-Trujillo and Treue, 2004), including appropriate peri-surgical analgesia and monitoring to minimize potential suffering. The German Primate Center has several staff veterinarians that regularly monitor and examine the animals and consult on any procedures. During the study the animals had unrestricted access to food and fluid, except on the days where data were collected or the animal was trained on the behavioral paradigm. On these days the animals were allowed unlimited access to fluid through their performance in the behavioral paradigm. Here the animals received fluid rewards for every correctly performed trial. Throughout the study the animals' psychological and medical welfare was monitored by the veterinarians, the animal facility staff and the lab's scientists, all specialized on working with non-human primates. The two animals were healthy at the conclusion of our study and were used in follow-up studies.

#### 2.1.2. Experimental procedure

Single unit action potentials were recorded extracellularly with single tungsten electrodes (FHC, Inc., Bowdoinham, ME, USA) after penetration of the dura with a sharp guide tube. The electrode was advanced using a hydraulic micropositioner (David Kopf Instruments, Tujunga, CA, USA). Impedances ranged from 0.5 to 2.8 MΩ. Neuronal activity was amplified and filtered (bandpass 150–5000 Hz). Action potentials in the majority of recorded units were sorted online using the Plexon data acquisition system (Plexon Inc., Dallas, TX, USA). In the first recording sessions action potentials were isolated using a window discriminator (BAK Electronics Inc., Mount Airy, MD, USA). Area MT was identified by its anatomical position, the high proportion of direction-selective cells, and the typical size-eccentricity relationship of RFs. Eye positions were monitored using a video-based eye tracking system (ET-49, Thomas Recording, Giessen, Germany). Eye positions were sampled at 230 Hz, digitized and stored at 200 Hz. Fixation was controlled during the recordings to stay within a window of 1.2° radius around the fixation spot.

#### 2.1.3. Visual stimuli

The experiments were conducted using an Apple Macintosh computer running custom software and a Sony Trinitron (22″) monitor with 75 Hz refresh rate. The monkey viewed the display binocularly in a dimly lit room from a distance of 57 cm. The spatial resolution of the display was 40 pixels per degree of visual angle. The shape of the RF, as well as its preferred direction and speed were estimated in a separate mapping and tuning session performed before the main task. The bidirectional stimuli were two RDPs presented within stationary adjacent virtual apertures matching the excitatory part of the RF (see Figure 1A). Another pair of RDPs was presented far outside the RF in the opposite visual hemifield symmetrically to the first pair with respect to the fixation point. Each RDP had a density of 10 dots per square degree. The width of each dot was 6 min of arc. All dots were white (luminance 85 cd/m^2^) and were displayed on a gray background (luminance 15 cd/m^2^). The basic speed of the dots in the RDP was matched to the preferred speed of the neuron, usually between 4 and 16°/s. The 12 directions of the patterns used to recover the tuning curve were chosen such that one of them was well-aligned with the preferred direction of the neuron.

See also Kozyrev et al. (under revision) for more details on monkey training and surgery, experimental procedures, and visual stimuli.

### 2.2. Data used for analysis

**Figure 3:**
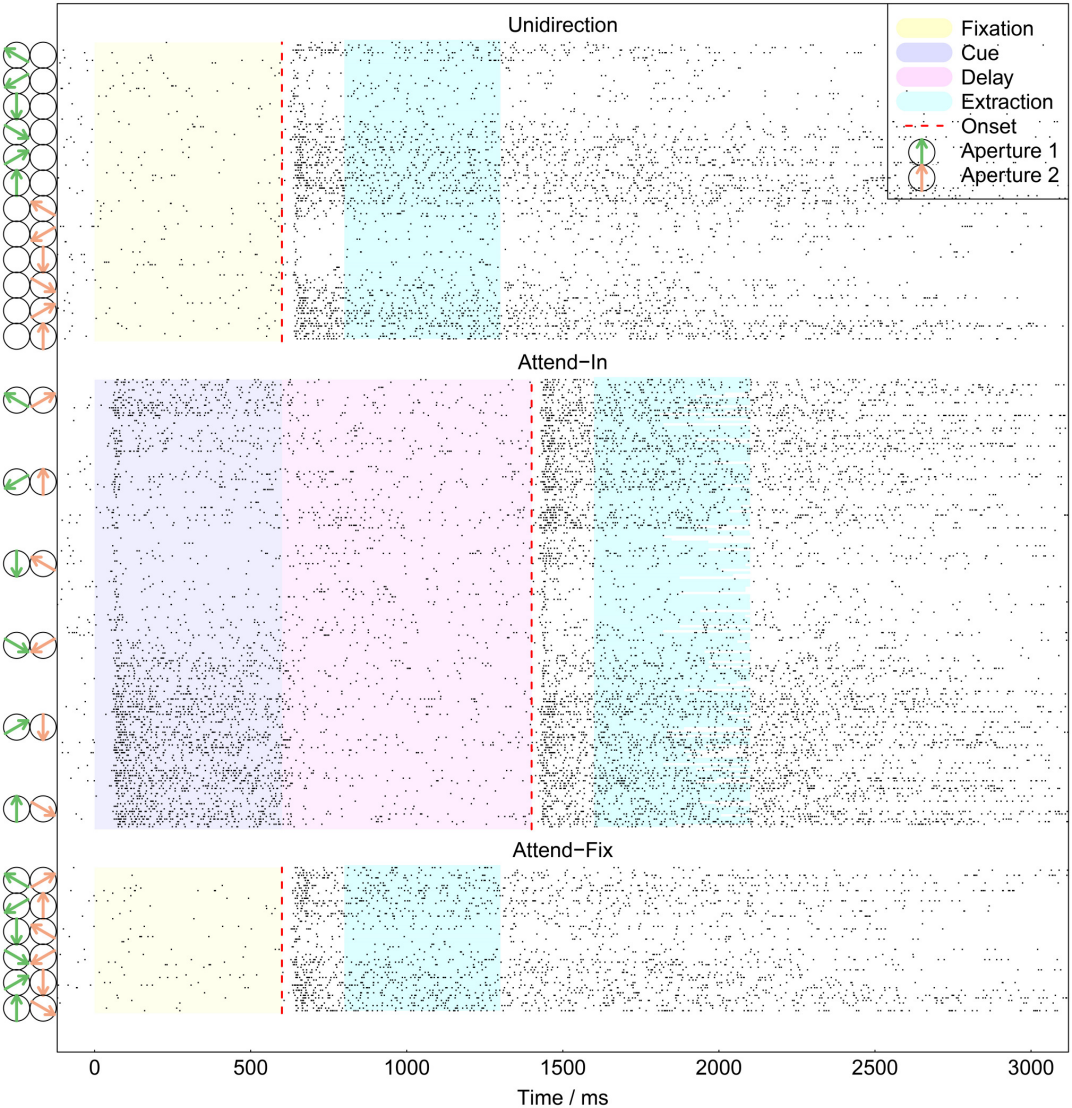
**All recorded spike trains from example neuron**. Spikes are shown as points. The extraction intervals indicate the data used for analysis. They varied in length between trials in the *attend-in* condition, in which the imperative change in the target RDP could happen <700 ms after the onset of the stimulus. At time 0 the monkey pressed a lever to start a trial. The dashed red lines indicate the onset of the target. To the left are shown the presented RDPs, for readability only every second stimulus is shown.

The recorded spike trains covered about the first 3000 ms of each trial. Figure 3 shows all spike trains from an example neuron. The periods of fixation, cue, delay, and intervals extracted for analysis are indicated with different colors. The onset of the target is indicated by the red dashed lines. Clear *delay* and *burst* effects are seen: When the RDP appears on the screen, the neuron has after a short delay a period of bursting behavior. We excluded the first 200 ms because of a large variability in the strength and length of the initial transient period around 50–200 ms. The latter was probably dependent on adaptation to the cue and other factors which are not considered by the relatively simple models we tested here. Thus, only the time interval from 200 to 700 ms after the onset of the RDPs were analyzed. Excluding the transient response epoch in the analysis is widely done, and this time window is also used by Katzner et al. (2009) and Martınez-Trujillo and Treue (2002) as the period where the MT neurons show robust attentional modulation. In case the speed or direction of motion of an RDP changed before 700 ms, the analysis interval terminated when the change occurred. We chose this interval for analysis in order to bypass the delay and burst periods and analyze an approximately constant firing rate.

In total 166 neurons have been recorded. However, we required at least two spike trains for each condition to include a neuron into further analysis, which resulted in 109 analyzed neurons. Summary statistics on number of trials and neurons can be found in Table 1. In an *attend-out* condition, the target always moved in either the preferred or the null direction of the recorded neuron, and the stimulus in the RF always moved in the preferred direction. Accordingly, the results from the attend-out condition could not be analyzed on a par with results from the other conditions. These data were therefore discarded. Regarding behavioral performance, we only included trials where the monkey detected the transient change and responded correctly (see Experimental procedures).

**Table 1:** **Summary statistics of sample sizes**.

	**Number of combinations**	**Quantiles of number of trials**				
**Condition**		**Min**	**10%**	**50%**	**90%**	**Max**
	Neurons × stimuli					
*fix1*	84 × 12	2	3	4	6	7
*fix2*	84 × 12	2	3	4	6	7
*attend-fix*	109 × 12	2	3	4	7	16
*attend-in*	109 × 12	3	8	12	18	31
	Neurons					
*fix1*	84	25	34	48	69	84
*fix2*	84	25	34	48	70	84
*attend-fix*	109	25	36	55	85	186
*attend-in*	109	61	90	138	207	272

*The neurons measured in conditions fix1 and fix2 are a subset of the neurons measured during the other two conditions*.

### 2.3. Notation

Index *d* indicates the 12 directions: $d\in \left\{0,\frac{\pi }{6},\frac{2\pi }{6},\ldots ,\frac{11\pi }{6}\right\}$, and *l* ∈ {1, 2} indicates (the location of) the stimulus, which is either aperture 1 or aperture 2. The index c∈C indicates the experimental condition; C = {*attend-fix, attend-in, fix1, fix2*}. In condition *fix1*, the unidirectional RDP appears in aperture 1, and in *fix2*, the RDP appears in aperture 2. Consider the time interval (0, *T*] extracted for analysis, where for simplicity we set the start point 200 ms after onset of the stimulus to be at time 0, and thus *T* ≤ 500 ms. The interval contains a sequence of *N* spikes: 0 < *t*_1_ < *t*_2_ < ⋯ < *t*_*N*_ < *T*, where *t*_*i*_ is the time of occurrence of the *i*th spike. We write τ = (0, *t*_1_, *t*_2_, …, *t*_*N*_, *T*), and *N*(*t*) denotes the number of spikes that occurred in the time interval (0, *t*] for 0 < *t* ≤ *T*.

### 2.4. Data analysis

The spike trains were modeled as stochastic point processes (Truccolo et al., 2005; Kass et al., 2014, chap. 19). The conditional intensity function (Daley and Vere-Jones, 1988) of a general point process model is defined by

(1) $$\begin{array}{l} \lambda \left(t|H_{t}\right)=\underset{\Delta t\to 0}{\lim}\frac{Pr\left(N\left(t+\Delta t\right)-N\left(t\right)=1|H_{t}\right)}{\Delta t}, \end{array}$$

where *H*_*t*_ denotes the spike history up to time *t*. Then λ(*t*|*H*_*t*_)Δ*t* approximates the probability of observing a spike in (*t, t* + Δ*t*] for Δ*t* small.

The likelihood of observing spike train τ is (Daley and Vere-Jones, 1988; Kass et al., 2014)

(2) $$\begin{array}{l} L\left(\tau ;\theta \right)=\left[\overset{N}{\underset{i=1}{\prod }}\lambda \left(t_{i}|H_{t_{i}};\theta \right)\right]\exp\left\{-\int_{0}^{T}\lambda \left(s|H_{s};\theta \right)ds\right\} \end{array}$$

where θ is a vector of model specific parameters, which should be estimated from data. The parameter vector θ for the two models will be specified in Section 2.5. In practice the measurements of the spike times are discrete, indicating whether or not they occur in time intervals of length Δ*t* = 1 ms, where Δ*t* is so small that it contains at most one spike and the conditional intensity function can be assumed constant within each interval. We approximate the integral in Equation (2) by a discrete sum and obtain

(3) $$\begin{array}{r} L\left(\tau ;\theta \right)\approx \left[\overset{N}{\underset{i=1}{\prod }}\lambda \left(t_{i}|H_{t_{i}};\theta \right)\right]\;\exp\;\left\{-\overset{\frac{T}{\Delta t}}{\underset{n=1}{\sum }}\lambda \left(n\Delta t|H_{n\Delta t};\theta \right)\Delta t\right\}. \end{array}$$

Truccolo et al.‘ modeled the spike train as a discrete sequence of conditional Bernoulli events, and obtained the same result as Equation (3) through probability mass functions (Truccolo et al., 2005).

Spike trains from different trials are assumed independent, and the likelihood of the entire data set will therefore be the product of individual likelihoods of the form (equation 2). Parameters are assumed constant for all trials from a neuron, but can differ from neuron to neuron. The estimation can therefore be done individually for each neuron. For each neuron, the likelihood of the recorded spike trains was computed by use of the conditional intensity function assuming, in turn, the response-averaging and the probability-mixing models.

The *conditional intensity function* is modeled with three components: (1) a base firing rate, *r*_*l*_, computed using Gaussian tuning curves (see below), which describes the effect of stimulus *l* and its direction of movement; (2) a scaling function depending on time, *a*(*t*); and (3) the effects of the spike history, *h*(*H*_*t*_). It is assumed to be of the following form:

(4) $$\begin{array}{l} \lambda \left(t|H_{t}\right)=r_{l}\exp\left\{a\left(t\right)+h\left(H_{t}\right)\right\}. \end{array}$$

The trend in the firing rate is modeled linearly (Cox and Lewis, 1966), *a*(*t*) = γ_0_*t*, where γ_0_ is a parameter. Since the firing rate decreases over time, γ_0_ is expected to be negative.

For the history component we use linear addition of the spikes in the past *m* time units:

(5) $$\begin{array}{l} h\left(H_{t}\right)=\overset{m}{\underset{i=1}{\sum }}\gamma_{i}\Delta N_{t-i\Delta t}, \end{array}$$

where Δ*N*_*t*_ ∈ {0, 1} denotes whether or not there is a spike in the interval [*t, t* + Δ*t*). Parameter γ_*i*_ is a spike response weight and quantifies the effect of having a spike *i* steps back in time. If it is negative, the effect is inhibitory, if it is positive it is excitatory. In the data analysis, *m* = 10 has been used. We have repeated the analysis with other memory lengths, but for larger *m*, the estimates of γ_*i*_ were close to zero, and the estimates of other parameters were stable, not changing the conclusions from the analysis, see **Figure 9F**.

The final model for the conditional intensity function used in the analysis is thus:

(6) $$\begin{array}{l} \lambda \left(t|H_{t}\right)=r_{l}\exp\;\left[\gamma_{0}t+\overset{10}{\underset{i=1}{\sum }}\gamma_{i}\Delta N_{t-i\Delta t}\right]. \end{array}$$

A *Gaussian tuning curve* is used to model the firing rate *r* as function of direction of motion *d*, with mean in the preferred direction, *D*, of the neuron. The preferred direction was estimated in a separate mapping and tuning session performed before the main task. For simplicity, we therefore set *D* = 0, and measure the direction of the stimulus RDP in deviation from the preferred direction. Since the stimulus is a direction (an angle), the rate function should be periodic with period 2π, and we apply the method given by Shokhirev et al. (2006), see also Treue S. and Trujillo J. (1999). For a neuron responding to stimulus *l* moving in direction *d*, the firing rate is given by

(7) $$\begin{array}{l} r_{l}=f\left(d|A_{l},\sigma_{l},r_{0}\right)=A_{l}\exp\;\left[-\frac{∥d-D{∥}_{2\pi }^{2}}{2\sigma_{l}^{2}}\right]+r_{0}, \end{array}$$

where *A*_*l*_ denotes the amplitude (directional gain), σ_*l*_ denotes the standard deviation (selectivity of the preferred direction), and *r*_0_ is the spontaneous firing rate in absence of a stimulus. The first two depend on the stimulus. The function ∥ *d* − *D* ∥ _2π_ = mod(*d* − *D* + π, 2π) − π ensures that the firing rate is periodic and symmetric around *D*. Figure 2 shows the mean firing rates fitted by Gaussian tuning curves. The unidirectional cases are modeled by single Gaussian curves, and the bidirectional cases are modeled by a mixture of two Gaussian curves. Along the *x*-axis, an upward arrow indicates the preferred direction. The insert illustrates the periodic function.

#### 2.4.1. Stimulus weights in bidirectional conditions

In the *attend-fix* condition two RDPs are shown in the RF, one in aperture 1 and one in aperture 2. The neuron may favor one location over the other, which is modeled by assigning a weight to each location. These weights will be modified in the *attend-in* condition, where the weight to the attended location is expected to increase. Let *w*_*c, l*_ denote the weight of stimulus *l* under a bidirectional experimental condition *c*, such that *w*_*c*_ = *w*_*c*, 1_ + *w*_*c*, 2_ denotes the sum of the weights. Let *p*_*c*_ = *w*_*c*, 1_/*w*_*c*_ and 1−*p*_*c*_ = *w*_*c*, 2_/*w*_*c*_ denote the normalized weights.

#### 2.4.2. Attentional scaling parameters

In the *attend-in* condition, a prior cue shows a replica of the stimulus to be attended (stimulus 1) including its location and direction of movement. The cue causes a multiplicative increase in the rate of firing in response to the cued stimulus (the stimulus in aperture 1). Sometimes the cue also changes the rate of firing in response to the uncued stimulus (stimulus 2). We use a scaling parameter *a*_*l*_ multiplying the amplitude *A*_*l*_ to model such attentional effects for stimulus *l*. The resulting firing rate is *r*_*l*_ = *f*(*d*|*a*_*l*_*A*_*l*_, σ_*l*_, *r*_0_). Without loss of generality, the scaling parameter *a* may be assumed to have a value of 1 in conditions *attend-fix, fix1*, and *fix2*, in which directions of movement are irrelevant to the task to be performed by the monkeys.

### 2.5. Models

Let the rates of firing of the recorded cell be *r*_1_ and *r*_2_, respectively, when objects 1 and 2 are presented alone in the classical RF of the cell.

The *probability-mixing* model assumes a neuron responds to one and only one of the stimuli within its RF at a time, and the probability of responding to a particular stimulus depends on the weight of that stimulus. Hence, the probability that a neuron under a bidirectional experimental condition *c* reacts to stimulus *l* is given by *p*_*c*_. Thus,

(8) $$\begin{array}{l} r=\{\begin{array}{l} r_{1},\text{ with probability }p_{c}\\ r_{2},\text{ with probability }1-p_{c} \end{array}, \end{array}$$

where *r*_*l*_ is given by Equation (7), except that *A*_*l*_ is substituted by *a*_*l*_*A*_*l*_ in the *attend-in* condition. The likelihood of all data from one neuron is then

(9) $$\begin{array}{r} L\left(\theta \right)=\underset{c\in C}{\prod }\overset{12}{\underset{k=1}{\prod }}\overset{m_{c,k}}{\underset{j=1}{\prod }}\left(p_{c}L\left(\tau_{c,k,j};\theta_{1}\right)+\left(1-p_{c}\right)L\left(\tau_{c,k,j};\theta_{2}\right)\right), \end{array}$$

where *p*_*c*_ = 1 in the *fix1* condition, *p*_*c*_ = 0 in the *fix2* condition, the individual likelihoods *L*(·;·) are given by Equation (2), and θ_*l*_ contains the stimulus specific parameters (*A*_*l*_, σ_*l*_, *a*_*l*_) besides the common parameters (*r*_0_, *p*_*attend*−*in*_, *p*_*attend*−*fix*_, γ_0_, γ_1_, …, γ_10_). Thus, θ contains all 20 parameters. Here τ_*c, k, j*_ denotes the spike train under the *c*th condition, *k*th direction and *j*th trial, where *m*_*c, k*_ is the number of trials under the specific experimental condition.

A numeric overflow issue arises when computing the log likelihood, since it may contain the logarithm of the sum of two small numbers, log(δ_1_ + δ_2_). This happens especially when the spike train is long, and the current parameters in the optimization algorithm are far from the optimal ones. We apply the *log-sum-exp* formula (Press, 2007): $\log\left(\delta_{1}+\delta_{2}\right)=\log^{*}\delta +\log\left(e^{\log\delta_{1}-\log^{*}\delta }+e^{\log\delta_{2}-\log^{*}\delta }\right)$, where $\log^{*}\delta =\max\left(\log\delta_{1},\log\delta_{2}\right)$.

The *response-averaging model* assumes the firing rate to be a weighted average rate over all stimuli,

(10) $$\begin{array}{r} r=p_{c}r_{1}+\left(1-p_{c}\right)r_{2}. \end{array}$$

The likelihood is

(11) $$\begin{array}{r} L\left(\theta \right)=\underset{c\in C}{\prod }\overset{12}{\underset{k=1}{\prod }}\overset{m_{c,k}}{\underset{j=1}{\prod }}L\left(\tau_{c,k,j};\theta \right). \end{array}$$

The number of parameters in the response-averaging model is one less than the probability-mixing model, because in the *attend-in* case not all three parameters (*p*_*attend*−*in*_, *a*_1_, *a*_2_) can be identified. We define *b*_1_ = *p*_*attend*−*in*_*a*_1_ and *b*_2_ = (1 − *p*_*attend*−*in*_)*a*_2_. In Table 2 the parameters entering in θ for the two models are summarized.

**Table 2:** **Parameters entering the parameter vector θ of the two models**.

**Model**	**Parameter**	**Explanation**
Common	γ_0_	Decay constant
	(γ_1_, γ_2_, …, γ_10_)	Spike response weights
	(*A*_1_, *D*_1_, σ_1_)	Parameters for the tuning curve of stimulus 1
	(*A*_2_, *D*_2_, σ_2_)	Parameters for the tuning curve of stimulus 2
	*r*_0_	Spontaneous firing rate
	*p*_*attend*−*fix*_	Probability/weight of stimulus 1 in *attend-fix*
Probability-mixing	*p*_*attend*−*in*_	Probability of stimulus 1 in *attend-in*
	*a*_1_	Attentional scaling of stimulus 1
	*a*_2_	Attentional scaling of stimulus 2
Response-averaging	*b*_1_ = *p*_*attend*−*in*_ · *a*_1_	Identifiable parameter for stimulus 1
	*b*_2_ = (1−*p*_*attend*−*in*_)·*a*_2_	Identifiable parameter for stimulus 2

In the unidirectional conditions, the response-averaging model and the probability-mixing model make the same predictions, and the firing rate is given by Equation (7). In the bidirectional conditions, the predictions of the two models differ as follows. In the *response-averaging* model, the firing rate to a stimulus pair in the *attend-fix* condition is a weighted average of the responses (firing rates) obtained to the individual stimuli in the unidirectional conditions (given by equation 7). However, the firing rate to a stimulus pair in the *attend-in* condition is a weighted average of *scaled* versions of the responses to the individual stimuli in the unidirectional conditions, where the scaling factor (*gain factor*) for a stimulus varies with the location of the stimulus (aperture 1, which showed the stimulus to be attended, vs. aperture 2, which showed a stimulus to be ignored). In the *probability-mixing* model, the firing rate to a stimulus pair in the *attend-fix* condition is a probability mixture of the responses (firing rates) to the individual stimuli in the unidirectional conditions. The firing rate to a stimulus pair in the *attend-in* condition is a probability mixture of scaled versions of the responses to the individual stimuli in the unidirectional conditions, where the scaling factor (gain factor) for a stimulus again varies with the location of the stimulus (aperture 1 vs. aperture 2).

#### 2.5.1. Diagnostic neurons

Whereas, some neurons are highly diagnostic in distinguishing between the response-averaging and the probability-mixing model when a certain pair of stimuli is presented in apertures 1 and 2 (see Figure 1A), responses of other neurons cannot be used for distinguishing between the two models. One example of a neuron that fails to distinguish between the models is a neuron that almost always responds as if only the stimulus in aperture 1 is present. Such a neuron behaves (to an arbitrarily good approximation) in accordance with a response-averaging model in which the response to the stimulus in aperture 1 is weighted much stronger than the response to the stimulus in aperture 2. At the same time, the neuron behaves in accordance with a probability-mixing model in which the probability of responding to the stimulus in aperture 1 is nearly 1. This, however, does not mean aperture 2 is not inside the RF, since the neuron does respond when a single stimulus is present in either aperture 1 or 2 alone. The above example occurs if one stimulus has a much stronger attentional weight than the other.

Another example of a neuron that cannot be used for distinguishing between the two models is a neuron in which the rate of firing is nearly the same for the stimulus in aperture 1 as for the stimulus in aperture 2. Regardless of the distribution of weights across the two stimuli, the neuron behaves in accordance with both a response-averaging model (averaging equals single firing rates) and a probability-mixing model (mixing equals single firing rates). In our experimental setup this is never the case, since the bidirectional stimuli always differ with 120, and for all neurons there are trials where this difference force firing rates to be different, as seen from the Gaussian tuning curves in Figure 2.

**Figure 4:**
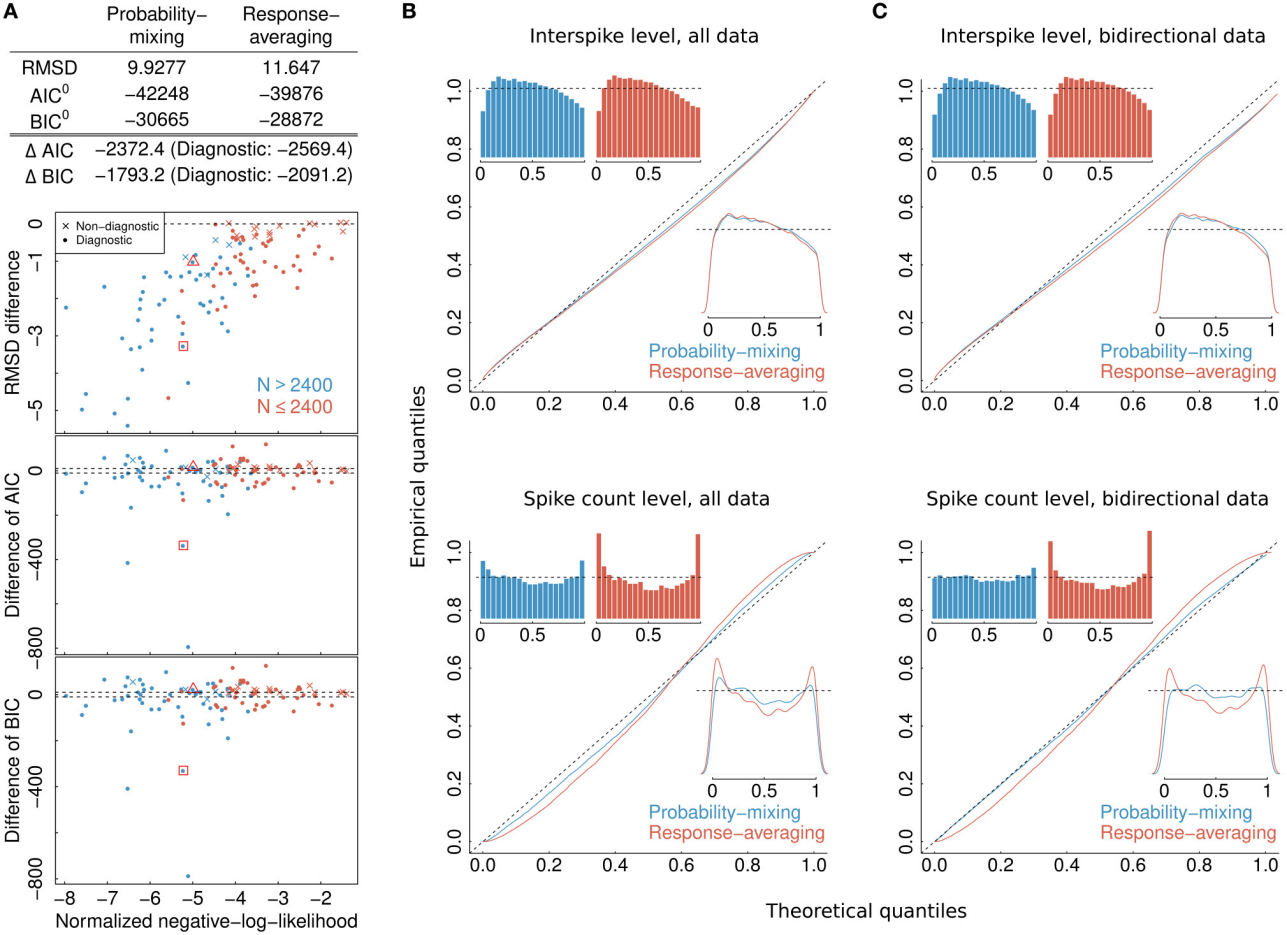
**Model selection and model checking**. **(A)** Differences in BIC, AIC, and RMSD values between the probability-mixing model and the response-averaging model (the former minus the latter). In all three cases, a smaller value means a better fit, so negative differences favor the probability-mixing model, whereas positive differences favor the response-averaging model. The squared and the triangled points are the example neurons from Figure 1B. The table on top provides the total saturated AIC, saturated BIC and RMSD values for each model. **(B)** QQ-plots of uniform residuals on interspike level (top) and on spike count level (bottom) for both models based on all observed data. The inserts are histograms (top) and density plots (bottom) of the uniform residuals. **(C)** The same as in **(B)** but calculating the uniform residuals on only bidirectional data.

Examples of neurons that are highly diagnostic in distinguishing between the response-averaging and the probability-mixing model are neurons with close to equal weighting of stimuli in apertures 1 and 2 but very different responses to the two stimuli. Figure 1B exemplifies the expected behavior of such neurons according to the probability-mixing model and according to the response-averaging model, respectively. Figure 1C shows histograms of empirical firing rates of the corresponding spike trains in Figure 1B. As can be seen, according to the probability-mixing model, the neuron responds either to stimulus one or to stimulus two, which generates a wide variation in firing rates (bimodal distribution). In contrast, by the response-averaging model, the responses to stimulus pairs all have similar rates (unimodal distribution). We defined a *diagnostic neuron* based on the estimated probabilities (in the probability-mixing model) or the weights (in the response-averaging model). These two example neurons are indicated by a square and a triangle, respectively, in Figure 4. We call a neuron diagnostic if either the two *p*_*attend*−*fix*_ estimates from the two models both are between 0.2 and 0.8, or if *p*_*attend*−*in*_ in the probability-mixing model fulfills the same criterion. This provides 90 diagnostic neurons, out of the 109 analyzed neurons. All analyses were performed on the entire data set, but where relevant, we indicate partial results only including the diagnostic neurons, and we highlight the type of neuron in the figures.

Note that whether a neuron is diagnostic or not does not reflect how well the models fit the data of that neuron. It only indicates that diagnostic neurons behave differently under the two models, whereas non-diagnostic neurons behave similarly under the two models, and contain little information for model selection.

#### 2.5.2. Relation of probability-mixing model to NTVA

The probability-mixing model is closely related to the Neural Theory of Visual Attention (NTVA) (Bundesen et al., 2005; Bundesen and Habekost, 2008). Attentional weights and the ways they are computed and used are the same in the probability-mixing model as in NTVA. In particular, in both the probability-mixing model and NTVA, the probability that an MT neuron represents an object *x* in its classical receptive field equals the attentional weight of object *x* divided by the sum of the attentional weights of all objects in the receptive field of the neuron. Also, the nature of attentional weights is the same in the two models. Thus, in both models, the attentional weights may depend on many different features of the objects, including features computed in areas other than MT.

Consider a trial in which an MT neuron that prefers motion in direction *D* represents a stimulus *l*, moving in direction *d*. Given that the neuron represents stimulus *l*, it responds as though stimulus *l* were the only object in its receptive field. By the rate equation of NTVA, the activation of the neuron, *v*(*l, D*), equals the product of the strength of the sensory evidence that stimulus *l* moves in direction *D*, η(*l, D*), and the bias in favor of seeing movement in direction *D*, β_*D*_. In the current article, among others (Bundesen and Habekost, 2008), a base rate, *r*_0_, is effectively added to the product of η(*l, D*) and β_*D*_. Thus, according to NTVA,

(12) $$\begin{array}{l} v\left(l,D\right)=\eta \left(l,D\right)\beta_{D}+r_{0}, \end{array}$$

where η(*l, D*) may be given by

(13) $$\begin{array}{l} \eta \left(l,D\right)=A_{l}\exp\;\left[-\frac{∥d-D{∥}_{2\pi }^{2}}{2\sigma^{2}}\right] \end{array}$$

as suggested by Equation (7). By NTVA, η(*l, D*) is independent of attention, but the bias parameter β_*D*_ depends on the attentional condition. In conditions *attend-fix, fix1*, and *fix2*, directions of motion are task-irrelevant, whence β_*d*_ (a measure of the importance of seeing motion in direction *d*) is a small number, say, β_0_, for all directions *d*. In condition *attend-in*, however, the stimulus in aperture 1 moves in the cued direction, whence β for its actual motion direction (= the cued direction) has a large value (say, β_1_). Thus, the categorization that the stimulus in aperture 1 moves in the cued direction is supported by both sensory evidence and perceptual bias. By contrast, in the same condition, the stimulus in aperture 2 moves in a direction that diverges from the cued direction by 120°, whence β for its actual motion direction has a smaller value (say, β_2_).

By Equations (12) and (13), the predicted firing rates remain constant if all β values are multiplied by a positive constant *k* while all amplitude parameters *A*_*l*_ are divided by the same constant *k*. Accordingly, without loss of generality, β_0_ can be set to a value of 1 if (i) β_1_ and β_2_ are changed in direct proportion to β_0_ and (ii) amplitude parameters *A*_1_ and *A*_2_ are changed in inverse proportion to β_0_. After these rescalings, the resulting values of β_1_ and β_2_ can be identified with scaling parameters *a*_1_ and *a*_2_, respectively. That is, *a*_1_ = β_1_/β_0_ and *a*_2_ = β_2_/β_0_.

Finally, we can extend NTVA to account for effects of presentation time *t* and spike history *H*_*t*_ by letting *v*(*l, D, t*|*H*_*t*_) be the conditional intensity function for a spike train and assuming that

(14) $$\begin{array}{l} v\left(l,D,t|H_{t}\right)=v\left(l,D\right)\;\exp\;\left[\gamma_{0}t+\overset{10}{\underset{i=1}{\sum }}\gamma_{i}\Delta N_{t-i\Delta t}\right], \end{array}$$

where *v*(*l, D*) is given by Equation (12).

In the suggested interpretation, the cue shown in the *attend-in* condition cues a particular direction of motion to be attended by pigeonholing (i.e., by setting β high for this direction) (Bundesen et al., 2005; Bundesen and Habekost, 2008). In addition to being used for pigeonholing, the cue can also be used for filtering (Bundesen et al., 2005; Bundesen and Habekost, 2008), in particular, filtering by location (by giving high attentional weight to stimuli that are located in aperture 1) and/or filtering by direction of motion (giving high attentional weight to stimuli that are moving in a particular direction).

### 2.6. Model selection by relative goodness of fit and cross-validation

The main aim of our article is to compare the abilities of the probability-mixing and the response-averaging models to explain the data. To select the best-fitting model, we use the Bayesian Information Criterion (BIC) and the Akaike information criterion (AIC), which compare likelihood values correcting for the number of parameters (Burnham and Anderson, 2002). Since only the difference of AIC (BIC) can be used for model comparison (Burnham and Anderson, 2002; Claeskens and Hjort, 2008), we subtract out the null deviance from the AIC (BIC) values for both models while preserving the difference. The null deviance is defined by −2 log(*L*^0^), where *L*^0^ is the likelihood value of the null model assuming that all spike trains from one neuron have the same firing rate. Given the two models, the weight in favor of the model with the lowest AIC (BIC) value is given by 1/(1 + exp(−Δ/2)) (Burnham and Anderson, 2002; Claeskens and Hjort, 2008), where Δ is the difference between the two AIC (BIC) values, and the weight in favor of the model with the highest value is given by exp(−Δ/2)/(1 + exp(−Δ/2)). Heuristically, the weight can be interpreted as the probability of the model to be the best among the considered models, in the sense of Kullback-Leibler information loss (Burnham and Anderson, 2002; Claeskens and Hjort, 2008).

This approach of statistical model selection to determine the most plausible model, each offering opposing biological explanations, using advanced statistical point process models to analyze single spike trains instead of trial-averaged responses, was also employed recently in Latimer et al. (2015). Here they determine whether firing rates during decision-making in the macaque lateral intraparietal area are gradually accumulating evidence toward a decision threshold, or whether decisions are taken as instantaneous jumps in the firing rates.

Model selection was done on individual neurons. However, assuming that the neurons we tested accomplished the same kind of processing but were statistically independent, the overall likelihood in favor of the probability-mixing and the response-averaging model, respectively, equals multiplication of the likelihoods of all of the individual neuron, or equivalently, summation of log-likelihood values, corresponding to summation of AIC (and approximately summation of BIC) values. We therefore also obtained overall AIC (BIC) values for the two models from the overall likelihoods, the numbers of parameters summed across all neurons, and the sample sizes of the data.

In addition to AIC and BIC criteria, we use the root mean squared deviation (RMSD) between observed and predicted firing rates and uniformity tests for general goodness of fit.

*Empirical and theoretical firing rates* can be compared to judge the goodness of fit. A quantitative measure is the RMSD between empirical and predicted rates for all spike trains of a neuron:

(15) $$\begin{array}{l} \text{RMSD}=\sqrt{\frac{1}{K}\overset{K}{\underset{i=1}{\sum }}{\left(r_{i}-{\hat{r}}_{i}\right)}^{2}}, \end{array}$$

where *K* is the total number of spike trains. The empirical rate, *r*, is given by *r* = *N*/*T*, where *N* is the number of spikes, and *T* is the total time of the spike train. The theoretical rate, $\hat{r}$, was estimated by

(16) $$\begin{array}{l} \hat{r}=\frac{1}{T}\int_{0}^{T}\lambda \left(t|H_{t},\hat{\theta }\right)dt. \end{array}$$

In the probability-mixing model, stimulus decoding is first applied. Stimulus decoding in a mixture model is finding which stimulus, l^*^, the neuron is most probably responding to given a spike train and the estimated parameters. This is a classification problem, and solved by the stimulus that maximizes the posterior probability of *l* given the spike train τ and estimated parameters $\hat{\theta }$: $l^{*}={\text{argmax}}_{l}P\left(l|\tau ,\hat{\theta }\right)$. Thus, in Equation (16) the classified stimulus is used.

### 2.7. Model control by uniform residuals

#### 2.7.1. Uniformity test

A common method is to apply the time rescaling theorem (Brown et al., 2002; Haslinger et al., 2010). For a spike train τ, the transformations

(17) $$\begin{array}{l} Z_{i}=\int_{t_{i}}^{t_{i+1}}\lambda \left(s|H_{s}\right)ds \end{array}$$

for *i* = 1, 2, …, *N*−1 are exponentially distributed with rate parameter 1, and thus,

(18) $$\begin{array}{l} Z=\int_{0}^{T}\lambda \left(s|H_{s}\right)ds \end{array}$$

is the total time of a Poisson process with rate parameter 1 having *N* events. The above is true if and only if λ(*s*|*H*_*s*_) represents the true conditional intensity function. This provides uniformity tests both on interspike interval level: *F*_*exp*_(*Z*_*i*_|1) ~ *U*(0, 1), where *F*_*exp*_(*Z*_*i*_|1) is the exponential distribution function with rate 1, and on spike count level: *F*_*pois*_(*N*|*Z*) ~ *U*(0, 1), where *F*_*pois*_(*N*|*Z*) is the Poisson distribution function with parameter *Z*. In the latter case, the discrete distribution is approximated by the uniform distribution by taking the average value of *F*_*pois*_(*N*|*Z*) and *F*_*pois*_(*N*−1|*Z*).

Intuitively, if and only if the model correctly describes the observed neuronal behavior, providing the correct spiking probability at each discretized time step Δ*t*, the transformation Equation (18) is distributed as a standard Poisson process. We verify the similarity between the transformation and the standard Poisson process, by checking the uniform residuals calculated on the two levels described above against a uniform distribution, by Quantile-Quantile (QQ) plots and histograms. If QQ-plots fall close to the indentity line, it indicates that the model describes the true neuronal behavior well, as well as if histograms are standard uniform, i.e., it has approximately equal number of residuals within each bin in the interval (0, 1).

### 2.8. Unimodality tests

The response-averaging model predicts a unimodal distribution of firing rates, whereas the probability-mixing model predicts a multimodal distribution when the neuron is exposed to bidirectional stimuli and firing rates to unidirectional stimuli are different. The unimodality test is a statistical test for unimodality of an empirical distribution, i.e., whether the distribution shows a single mode or multiple modes. The dip test (Hartigan and Hartigan, 1985) is one method to perform the unimodality test. A significant *p*-value of a dip test rejects the hypothesis that there is a single mode and indicates multiple modes in the empirical distribution. Thus, we can perform the dip test as an empirical measure for the probability-mixing or the response-averaging model. We tried to employ dip tests to test for unimodality of a distribution on the firing rates, but the data are too sparse to provide useful information. One particular obstacle is that when estimating empirical firing rates (by spike counts) on discretized intervals, if these intervals are too narrow, only a few spikes or none will be present in most intervals. Then the empirical firing rates only take a few distinct values, repeated many times, and the test always turns out positive since the rates seem to follow a discrete distribution. If intervals are not narrow, there will only be a few data points, not enough for a test. Instead, as an auxiliary measure, we tested unimodality of the distribution of interspike intervals (ISIs). There is no reason to expect the ISI distribution to be unimodal, even if the distribution of firing rates is, since memory effects may create complex behavior in the distribution of ISIs. However, if a particular neuron does not show a multimodal ISI distribution while being exposed to a unidirectional stimulus, but the distribution changes to multimodal when bidirectional stimuli are presented, there is some indication that this multimodality could be caused by the bidirectional stimuli, supporting the probability mixing model.

## 3. Results

Our basic observations were sequences of action potentials (spike trains) emitted by individual MT neurons in the different conditions of the experiment in response to visual movement in different directions. Models were fitted to the spike train data by maximum likelihood estimation using numerical optimization algorithms. A global optimization with the dividing rectangles algorithm (Jones et al., 1993) was first performed, and the resulting estimates were then used as initial values for a local optimization with the Nelder-Mead simplex algorithm (Nelder and Mead, 1965), providing the final estimates. All parameters were estimated simultaneously.

### 3.1. Results from model selection by relative goodness of fit and cross-validation

To select one of the two models, we calculated the RMSD, AIC, and BIC values. The lower plots in Figure 4A shows, for each individual neuron, the difference between the AIC (BIC) value given the best-fitting probability-mixing model and the AIC (BIC) value given the best-fitting response-averaging model, with the color indicating neurons with many observed spikes (more than 2400 spikes, cyan) or few observed spikes (<2400 spikes, magenta; the spike counts include all spikes from the given neuron inside the observation windows in the four experimental conditions). Diagnostic neurons are indicated with dots, non-diagnostic neurons are indicated with crosses. Values below 0 favor the probability-mixing model, values above 0 favor the response-averaging model. The difference in AIC (BIC) values is plotted against the sum of the negative log-likelihood values from the two models normalized by number of spikes, such that data points to the left are more trustworthy (approximately coinciding with those with larger sample sizes). Two dotted lines are drawn at ±10, representing the difference value of 10. This is the value suggested in Burnham and Anderson (2002) as the critical value for the less plausible model to have essentially no support in the data compared with the better model. A few neurons (depicted near the bottom of the plot) seemed highly diagnostic in distinguishing between the response-averaging and the probability-mixing model. Many other neurons failed to distinguish between the two models (neurons with values near zero). This could be due to limited sample sizes, since the cyan neurons are more trustworthy with larger sample sizes, and indeed tend to fall below 0. Furthermore, as expected, the non-diagnostic neurons typically have values around 0.

The values resulting from analyzing all neurons together are shown as AIC^0^ (BIC^0^) in the table at the top of Figure 4A. These values can be interpreted as the explanatory evidence in the models compared to the null model (Harrell, 2001), see Section 2.5 for definition of the null model. Furthermore, the differences between the two AIC (BIC) values, ΔAIC (ΔBIC), are indicated in the same table, both for all neurons, and for diagnostic neurons only. The overall AIC and BIC values, aggregating all the information from individual neurons, are much smaller for the probability-mixing model than for the response-averaging model, so both the AIC and the BIC strongly favor the probability-mixing model. Indeed, both (absolute) differences are greater than Δ = 1000. Thus, given the two models, according to both the AIC and the BIC criteria, the weight in favor of the probability-mixing model is 1/(1 + exp(−Δ/2)) ≈ 1, and the weight in favor of the response-averaging model is exp(−Δ/2)/(1 + exp(−Δ/2)) ≈ 0, see Section 2.6.

The upper plot in Figure 4A shows the difference between the RMSD between observed and predicted firing rates for the best-fitting probability-mixing model and the RMSD for the best-fitting response-averaging model. The RMSD values were calculated using 10-fold cross-validation on spike trains of each neuron. For most of the neurons, the RMSD for the best-fitting probability-mixing model was smaller than the RMSD for the best-fitting response-averaging model, and this is particularly obvious for more trustworthy neurons, and for diagnostic neurons. The RMSD for all data for both models are shown in the top table. As the AIC and the BIC, the RMSD criterion also favors the probability-mixing model. The RMSD results are more consistently in favor of the probability-mixing model for all diagnostic neurons compared with the AIC and BIC results. Note the different perspectives of these model selection methods: RMSD measures the predictive accuracy while AIC (BIC) measures the information loss of the proposed model from the truth. We conclude that the probability-mixing model predicts behavior of independent trials better or at least as well as the response-averaging model on all neurons.

The overall conclusion is that the analysis supports the probability-mixing over the response-averaging model.

### 3.2. Results from model control by uniform residuals

**Figure 5:**
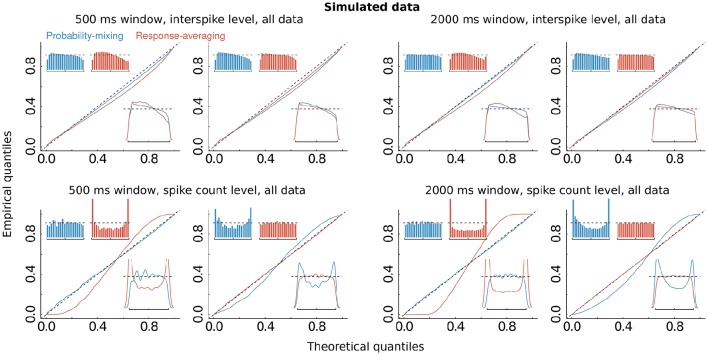
**Simulations**. The same as Figure 4B, but on simulated data, using the estimated parameters, and simulating from either of the two models, with observation windows of 500 ms (left panel with four figures) and of 2000 ms (right panel with the other four figures), respectively. Upper and lower panels show results at interspike and spike count levels, respectively. Panels in columns 1 and 3 show results for the data simulated from the probability-mixing model, panels in columns 2 and 4 show results for the data simulated from the response-averaging model.

The computations of AIC and BIC values show that the probability-mixing model fits the data better than does the response-averaging model, but neither information criterion tells us the absolute (as distinct from the relative) goodness of fit. For either model, goodness of fit to the spike trains of the neurons was evaluated by uniformity tests, both on interspike level and on spike count level (see Section 2). We merged all results based on Equation (17) from all spike trains of all neurons, to obtain uniform residuals on the interspike interval level, and all results based on Equation (18) to obtain uniform residuals on the spike count level. The uniform residuals were checked graphically in Figure 4B by histograms and QQ plots against the standard Uniform distribution. The histograms and plots of events at the interspike interval level show nearly the same goodness of fit for the probability-mixing model and the response-averaging model, but the histograms and plots of events at the spike count level show better fits for the probability-mixing model compared with the response-averaging model, as can be seen from the cyan QQ-plot being closer to the identity line, and cyan histograms being more uniform than the magenta ones in the lower plots. However, neither model is perfect. If a model is correct we expect the uniform residuals to lie on the identity line, which is not strictly the case for either one of the two models. We conjecture that this was partly caused by boundary effects inducing bias because the observation intervals were never longer than 500 ms. To check this, we conducted a simulation study, first simulating from both models using the estimated parameters, with observation intervals of both 500 and 2000 ms, and then estimating with both models (Figure 5). The interval of 2000 ms was chosen to be large enough for boundary effects to be negligible. The results suggested that the misfits could be explained, in part, by finite sample effects. Another feature not accounted for in the model is overdispersion, i.e., that the data show a larger variance than predicted by the model. This occurs for example if parameter values fluctuate from trial to trial, whereas the model assumes these constant. We therefore also plotted the uniform residuals using only the bidirectional data (Figure 4C), and the fit clearly improved, suggesting overdispersion.

**Figure 6:**
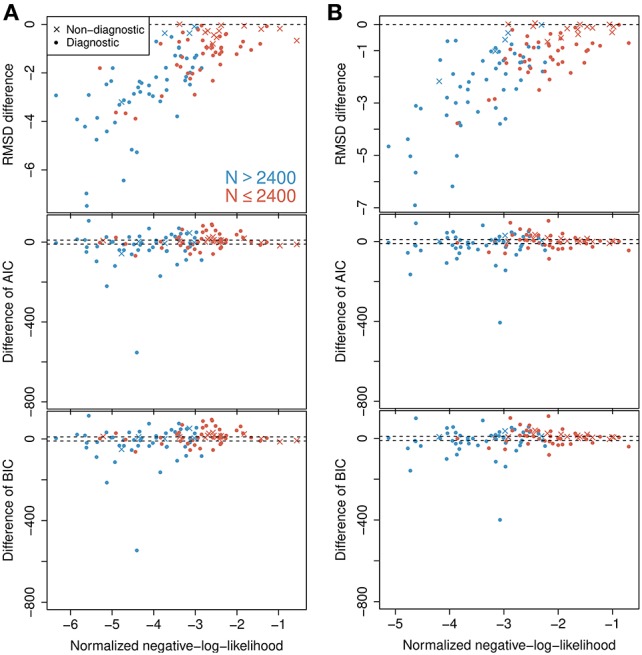
**Model selection on first and second halves of the observed time intervals**. Differences in BIC, AIC, and RMSD values between the probability-mixing model and the response-averaging model (the former minus the latter). **(A)** Analysis on first 250 ms of the observed time intervals (first half of the data). **(B)** Analysis on next 250 ms of the observed time intervals (second half of the data). Compare to Figure 4A for analysis of the full interval.

In the analysis it is implicitly assumed that under the probability-mixing model, the represented object does not change during the course of a trial of 500 ms. This is done to obtain more statistical validity, but might be questionable from a biological point of view. For example, Fiebelkorn et al. (2013) found that sustained attention naturally fluctuates with a periodicity of 4–8 Hz, with reweighting between different objects occuring at 4 Hz. To check the validity of using the full length of the 500 ms interval, we also tried splitting the data, reanalyzing separately on the first (0–250ms) and on the second (250–500 ms) halves. The analysis was conducted the same way as for the full 500 ms interval. The results on RMSD, AIC, and BIC are shown in Figure 6 for the first half (Figure 6A) and the second half (Figure 6B). There are only small and not relevant differences between the two halves for each criterion. At both halves, the RMSD favors the probability-mixing model, particularly for neurons with a large number of observations. The AIC and BIC also show similar distribution patterns between the two halves. A paired Wilcoxon signed-rank test was done for the differences ΔAIC at the first half against the second half with the null hypothesis being that ΔAIC does not change between halves. The obtained *p*-value is 0.858, implying no evidence of changes in ΔAIC. The test on ΔBIC gives *p* = 0.830, leading to the same conclusion. To summarize, the conclusions are essentially the same as for the full interval, and model fitting on the shorter intervals provide no extra information. Thus, we analyze the full 500 ms interval exploiting the entire data.

### 3.3. Results for unimodality tests

**Figure 7:**
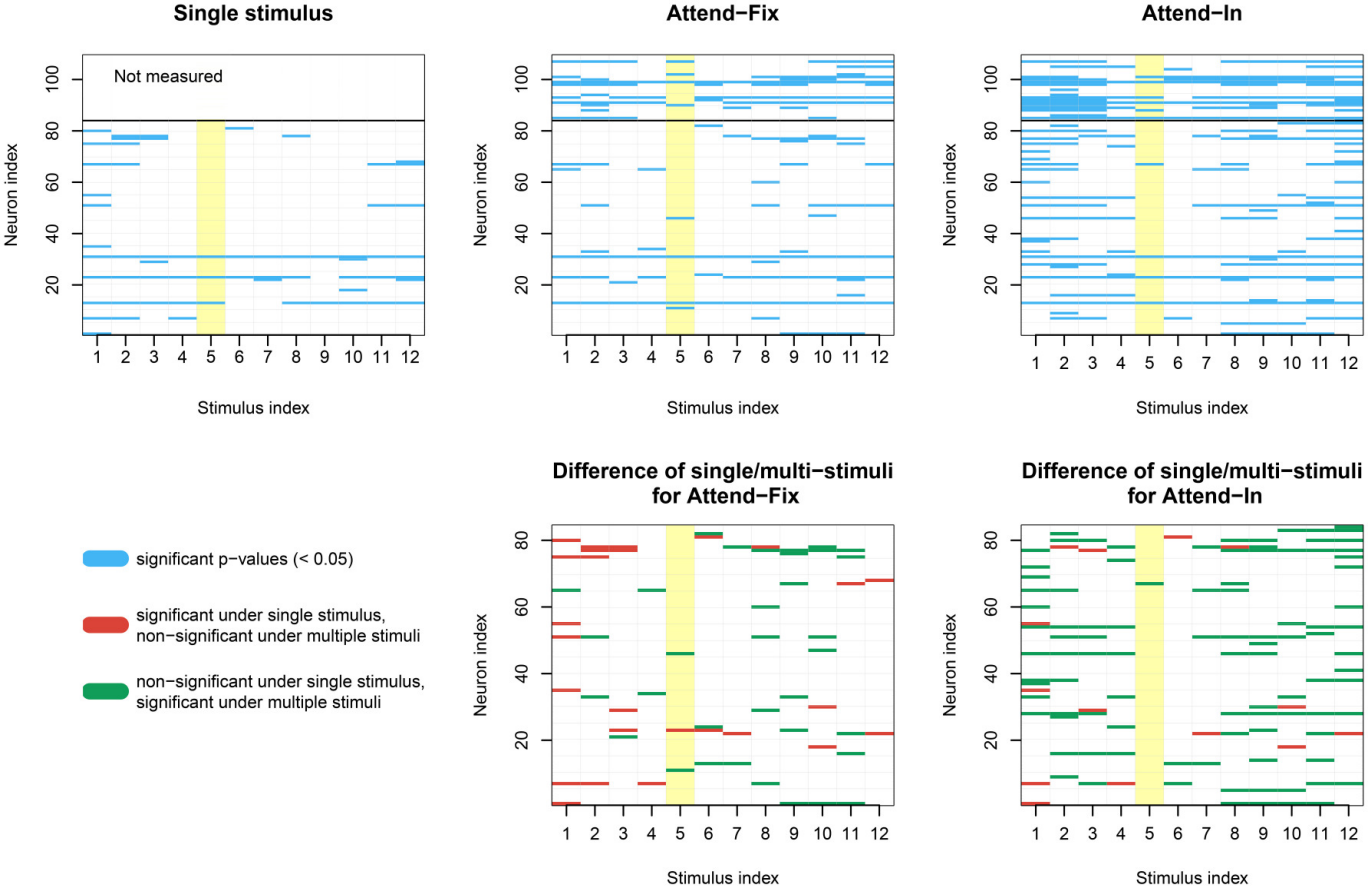
**Results of dip tests**. **(Upper panels)** Dip tests of each neuron at each condition for single stimulus trials (left panel) and stimulus mixture trials (middle and right panels) are illustrated. Each lattice point represents one test, and blue lattices are statistically significant (*p* < 0.05) against unimodality for the corresponding neuron and condition. The yellow lattice points are those corresponding to condition 5, where the stimulus in aperture 1 is 120° from the preferred direction, and the stimulus in aperture 2 is −120° from the preferred direction. This is the only condition where on average the firing rates for the two stimuli are equal, see the green and orange tuning curves on Figure 2, and thus, no bimodality is expected. **(Lower panels)** Changes from either significant to non-significant (red) or from non-significant to significant (green) *p*-values.

In Figure 7, dip tests of unimodality of the ISI distribution are illustrated for each neuron in each of the 12 direction-of-motion stimulus pairs. Each lattice point in the mesh figure represents one test, and blue lattice points (upper panels) show results that are statistically significant (*p* < 0.05) against unimodality (i.e., indicating at least two modes). In the upper left panel, data from the unidirectional stimulus conditions *fix1* and *fix2* are combined for the 84 neurons tested in these conditions. They were combined after normalizing by multiplying the ISIs by the average firing rate of the corresponding neuron and condition, so that the average firing rate of any neuron in any condition was 1. This was done in order not to observe an artificial bimodal distribution, caused by different response properties in aperture 1 and 2. Similar results are obtained by splitting in the two aperture conditions without normalization (results not shown). The bidirectional stimulus conditions were not normalized. Of the 1008 tests on unidirectional stimulus data, 6.05% were positive. This is close to the expected 5% from the coverage properties of the test, so it appears that under unidirectional stimulus, the ISI distributions are not multimodal. In the two upper panels to the right, data from the bidirectional stimulus *attend-fix* and *attend-in* are shown for the same 84 neurons (below the black line) and for the remaining 25 neurons (above the black line), which were not tested during unidirectional stimulus. Of the 1008 tests on bidirectional stimulus data from those neurons that were also tested in the unidirectional stimulus conditions, 6.8% (*attend-fix*) and 14.3% (*attend-in*) were positive. Including also the 25 neurons only tested in the bidirectional stimulus conditions, these numbers were 10.6 and 19.6% out of 1308 tests, respectively. Note that fewer significant lattice points appear in the *attend-fix* condition than in the *attend-in* condition, which is probably due to smaller sample sizes; see Table 2. The yellow lattice points are those corresponding to condition 5, where the stimulus in aperture 1 is 120° from the preferred direction, and the stimulus in aperture 2 is −120° from the preferred direction. This is the only condition where on average the firing rates for the two stimuli are equal, see the green and orange tuning curves on Figure 2, and thus, no bimodality is expected for most of the neurons in this condition. Indeed, in this case only 7.3 and 9.2% were significant. Condition 11, where the stimulus directions are ±60°, could also be expected to have equal firing rates for the two stimuli, and thus no multimodality, but since the firing rates are higher here, small differences in tuning curves for the two apertures result in large differences in firing rates, and thus, multimodality can still occur. In all three upper panels, most *p*-values are non-significant. However, compared with unidirectional stimulus conditions, more significant *p*-values appear in bidirectional stimulus conditions, mainly in condition *attend-in*, suggesting that stimulus plurality caused multimodality. This is illustrated in the lower panels, where changes from either significant to non-significant (red, 2.6% for *attend-fix*, 1.4% for *attend-in*) or from non-significant to significant (green, 3.4% for *attend-fix*, 9.6% for *attend-in*) *p*-values are indicated.

### 3.4. Population behavior of probability-mixing

In the probability-mixing model, a neuron attends to only one of a plurality of stimuli. A natural question is then whether in any given trial, individual neurons within a critical population behave consistently or independently. We therefore investigated correlations of nearby neurons. In the data, at most two neurons were recorded simultaneously, and there are 25 such neuron pairs. The two neurons do not necessarily have the same preferred direction of motion, but they differ at most 60° in their preferred direction. If neurons act consistently, we expect higher correlations for those pairs with the same preferred direction. We calculated the correlation of the firing rates of each neuron pair at different RDP-motion stimulus pairs using Spearman's correlation coefficient and Spearman's correlation test. Conditions *attend-in* and *attend-fix* are combined to make the sample size larger. The idea is that if two neurons have highly correlated attended stimuli, the correlation coefficient of rates will be large; otherwise, the coefficient will be near 0: Let two vectors *X* = (*X*_1_, *X*_2_, …, *X*_*n*_) and *Y* = (*Y*_1_, *Y*_2_, …, *Y*_*n*_) denote the firing rates of two neurons from *n* trials at a given stimulus pair. The corresponding *X*_*i*_ and *Y*_*i*_ are the firing rates of two neurons in the same trial *i*. Since there are two stimuli, *X*_*i*_ and *Y*_*i*_ could represent either stimulus. If the firing rates of the two neurons are positively correlated, then *X*_*i*_ and *Y*_*i*_ likely represent the same stimulus. If the firing rates are negatively correlated, then *X*_*i*_ and *Y*_*i*_ likely represent opposite stimuli. In both situations, non-zero correlation between *X* and *Y* is expected, assuming two stimuli generate sufficiently different firing rates. On the other hand, if the attended stimuli are not correlated, nor will the firing rates *X* and *Y* be correlated.

**Figure 8:**
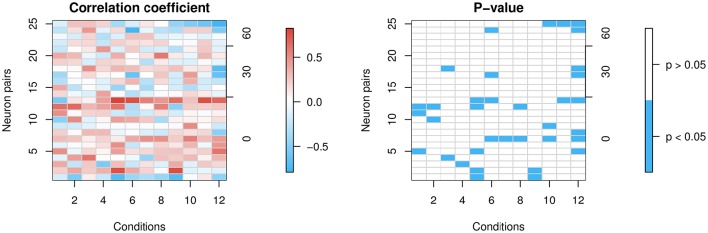
**Correlation of firing rates between neuron pairs**. The *x*-axes represent the 12 conditions and the left *y*-axes represent the 25 neuron pairs that are simultaneously recorded. Each lattice point in the mesh corresponds to one neuron pair at one condition. The 25 neuron pairs are ordered by the difference in degrees between the pair's preferred directions (0, 30, or 60°) shown in the right *y*-axes. If they differ in their preferred direction, then when one of the neurons is presented with its preferred direction, the other is not, and vice versa. So we expect less correlation in that case, whereas if they share the same preferred direction, there is more reason to believe they might be correlated. **(Left panel)** shows correlation coefficients. **(Right panel)** shows in blue significant *p*-values at a 5% level for the two-sided test of zero correlation.

The top left panel in Figure 8 shows the heat map of Spearman's correlation coefficients, and the top right panel shows the stronger positive correlations in red and stronger negative correlations in blue. The bottom left shows *p*-values from Spearman's correlation test for correlation being 0. The bottom right shows significant *p*-values in blue. The ratio of significant *p*-values over all 12 × 25 cells is 11.7%.

Most correlations are weak. However, we find a few stronger correlations in some neuron pairs, with a slight trend toward higher positive correlations for those with the same preferred direction, and negative correlations for those with differing preferred directions. The conclusions have to be interpreted with caution, though, since data on simultaneously recorded neurons are scarce.

### 3.5. Parameter estimates from maximum likelihood

**Figure 9:**
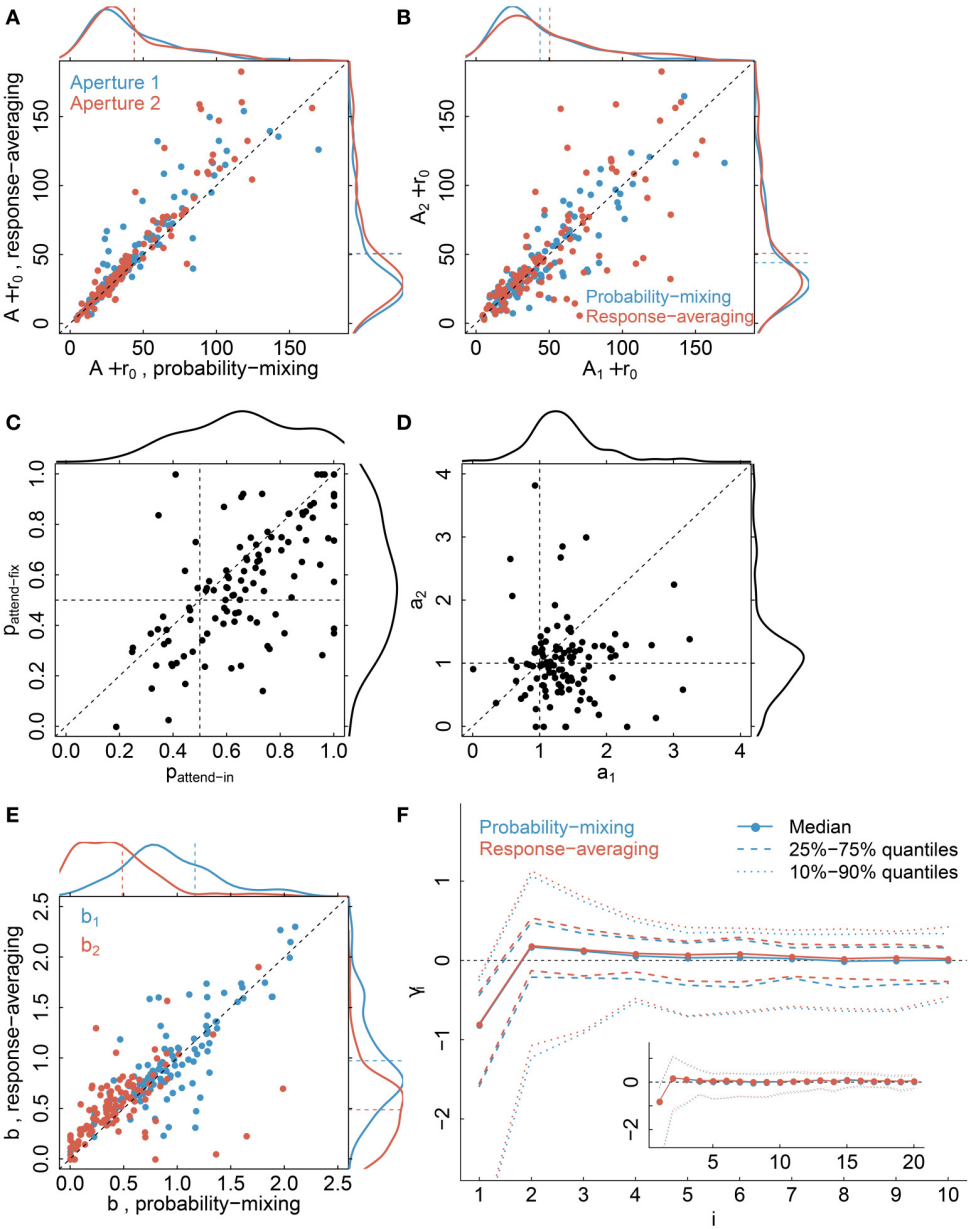
**Parameter estimates**. The plots compare the probability-mixing model with the response-averaging model and aperture 1 with aperture 2. **(A)** The sum of *A* and *r*_0_ from the response-averaging model is plotted against the probability-mixing model, using cyan for aperture 1 and magenta for aperture 2. The data densities are plotted on the top and on the right side, with dashed lines indicating the means. **(B)** We use the same estimates as in **(A)**, but plot aperture 2 against aperture 1, and use cyan for the probability-mixing model and magenta for the response-averaging model. **(C,D)** are only for the probability-mixing model, since these parameters are not all identifiable in the response-averaging model. **(C)** The probabilities of responding to aperture 1 in the *attend-fix* condition (*p*_*attend*−*fix*_) are plotted against *attend-in* condition (*p*_*attend*−*in*_). **(D)** Attentional effects for aperture 2 (*a*_2_) are plotted against aperture 1 (*a*_1_). **(E)** The identified parameters *b*_1_ and *b*_2_ in the response-averaging model are plotted against the corresponding values calculated from estimates in the probability-mixing model. **(F)** The medians of 10 spike response weight parameters from the conditional intensity function are plotted, together with the central 50 and 80% of the empirical distributions. We also fitted models with a memory of 20 ms, and the resulting estimates are plotted in the insert figure.

Parameter estimates, see Table 2 for a summary of model parameters, are illustrated in Figure 9 comparing the probability-mixing model with the response-averaging model and comparing aperture 1 with aperture 2. The upper panels, Figures 9A,B, provide the sum of *A* (directional gain) and *r*_0_ (firing rate without stimulus) from the Gaussian tuning curve, i.e., the maximal firing rate. The estimates from the probability-mixing model tend to be smaller than the estimates from the response-averaging model. Figures 9C,D cover only the probability-mixing model, because the weights and attentional scaling parameters are not identifiable in the response-averaging model. In Figure 9C the probabilities of responding to aperture 1 in the *attend-fix* condition (*p*_*attend*−*fix*_) are plotted against the probabilities of responding to aperture 1 in the *attend-in* condition (*p*_*attend*−*in*_). As expected, the probability of responding to aperture 1 is increased when attention is directed toward it, i.e., *p*_*attend*−*in*_ tends to be larger than *p*_*attend*−*fix*_ and also larger than 0.5. In Figure 9D attentional effects for aperture 2 (*a*_2_) are plotted against effects for aperture 1 (*a*_1_) in the *attend-in* condition. The effect of the cue is clearly detected: *a*_1_ tends to be larger than *a*_2_, and also larger than 1, i.e., attention increases the firing rate. In Figure 9E the identifiable parameters *b*_1_ and *b*_2_ in the response-averaging model are plotted against the corresponding values calculated from estimates in the probability-mixing model. Again, aperture 1 (*b*_1_) yields larger values than aperture 2 (*b*_2_), which is expected because of the cue. In Figure 9F the 10 spike response weight parameter estimates from the conditional intensity function are plotted. We use median values and quantiles. The first value γ_1_ is much more negative than the others, implying that a spike suppresses a spike in the next instance, corresponding to the refractory period. The spike response weight values decay to zero, illustrating the length of the memory of the spike history.

## 4. Discussion

Responses of sensory neurons to multiple presentations of identical stimuli can be highly variable (“cortical variability”; Goris et al., 2014; Cui et al., 2016). In this article we focus on one possible source of such cortical variability, namely, variation in which stimulus a sensory neuron responds to at a given time in a certain trial. Specifically, we aimed to determine if neurons in extrastriate visual cortex encode the presence of more than one distinct stimulus in their receptive field by alternating between response states, each predominantly representing one of the stimuli in the receptive field (Bundesen et al., 2005). We found evidence in support of such a multiplexing behavior by analyzing spike trains of individual trials (rather than average responses across trials) from neurons in visual cortical area MT of rhesus monkeys. Our approach is based on recent advances in statistics (chap. 19, Kass et al., 2014) that allowed us to distinguish responses from trial to trial. Employing statistical model selection using AIC, BIC, and RMSD, and model control using time rescaling and uniformity tests we find support for probability-mixing, i.e., serial switches between response states, distinct from the response-averaging suggested by pooling responses across multiple trials. Unimodality tests provide further support for multiplexing behavior by showing that stimulus plurality increases the probability of statistically significant multimodality of the interspike interval distribution.

For decades responses of sensory neurons in primate visual cortex have been investigated with single stimuli and their parametric variation. This has resulted in a very detailed understanding of the input-output-relationship of neurons in well-studied areas like primary visual cortex V1, area V4 along the temporal processing pathway and, most relevant for the current study, the middle-temporal area MT in the dorsal pathway.

More recently, particularly in MT, studies have focused on neuronal responses when multiple moving stimuli are present (spatially separated or in spatially coincident motion as transparent random dot patterns or sine wave gratings) in a given receptive field. Such studies have investigated “sensory” conditions, i.e., when none of the stimuli were behaviorally relevant (Snowden et al., 1991; Recanzone et al., 1997; Britten and Heuer, 1999; Treue et al., 2000; Majaj et al., 2007), as well as “attentional” conditions, i.e., task designs where one of the stimuli were behaviorally relevant (Seidemann and Newsome, 1999; Treue and Trujillo, 1999; Patzwahl and Treue, 2009; Niebergall et al., 2011a,b; Ni et al., 2012). All of these studies implicitly or explicitly assume that neurons always respond to multiple stimuli in their receptive field with a single response state that represents an integration (averaging with or without scaling or gain control) of the individual stimulus responses.

Here we successfully challenge this assumption by providing evidence for the ability of neurons to maintain distinct representations of the stimuli inside a given receptive field.

This ability to encode multiple stimuli by separate response states of individual neurons endows the visual system with a powerful feature, not present if the neurons combine the multiple stimulus responses into a common response. Indeed, once the responses have been averaged over all stimuli, reconstructing single stimuli from average responses at later stages of processing seems difficult if not impossible (Orhan and Ma, 2015). This is a core issue in understanding cortical representations of complex scenes, since they often have multiple stimuli placed in the same receptive field, particularly in the large receptive fields common in higher extrastriate cortical areas. If such neurons would integrate all stimuli inside their receptive field such “stimulus mixing” would severely compromise the brain's ability to maintain spatially detailed representations in natural vision (Orhan and Ma, 2015). The multiplexing we observe instead allows the information about which stimulus caused a particular neuronal response to be preserved and maintained across a series of processing stages from primary visual cortex through areas in extrastriate cortex.

Beyond this benefit, the temporal multiplexing of information provides a unique opportunity to selectively modulate the individual representations of the various stimuli contributing to a neuron's response. Such a reweighing has been suggested by models of attention since the perceptual effect of visual attention can often be described as an increase in the perceptual strength of attended stimuli at the expense of the perceptual strength of unattended stimuli.

One of these attention models, the Neural Theory of Visual Attention (NTVA; Bundesen et al., 2005), a neural interpretation of the mathematical Theory of Visual Attention (TVA; Bundesen, 1990), explicitly proposes that a neuron, when presented with a plurality of stimuli in its RF, responds to only one of them at a time. This hypothesis has not been tested before but was suggested by Bundesen et al. (2005) for computational and biological reasons (survival value), and it fits in with the way in which attentional modulations of sensory processing (in particular, so-called “filtering”) are explained in NTVA. In TVA stimulus representations race (compete) to become encoded into visual short-term memory (VSTM) before it is filled up. This race is influenced (biased) by attentional weights and perceptual biases, so that certain objects and features have higher probabilities of being perceived (encoded into VSTM). Thus the TVA presaged what later became known as the biased competition model of attention (Desimone and Duncan, 1995; Reynolds et al., 2000). Our data suggest that biased competition accounts of attentional responses need to be extended to allow for an alternation between response states rather than a single response state representing the outcome of the biased competition between the different stimulus representations.

The TVA is also compatible with the feature similarity gain model (Treue and Trujillo, 1999; Martinez-Trujillo and Treue, 2004). This model proposes that attention modulates brain activity by multiplicatively scaling neuronal responses with gain factors. The magnitude of a given gain factor represents the similarity between the stimulus preferences of the neuron and the currently attended features. In this model a selective enhancement or suppression of individual stimuli (based either on the stimulus' spatial location or its features Xue et al., 2016) is achieved on the population level because attention to a given feature increases the responses of all neurons preferring the same or similar features. In the TVA, the gain factor in question is the multiplicative perceptual bias toward feature *i* (β_*i*_), which is applied to neurons that are coding feature *i*. Incorporating the observed multiplexing into the feature similarity gain model would further elaborate the approach of the model to selective enhancement of attended features and locations.

Our observation that neuronal responses alternate between response states is reminiscent of the hypothesis that stimulus sampling under continuous attentional allocation follows a periodic process (Busch and VanRullen, 2010). While this potential link is intriguing, our data did not allow us to test the duration of individual response states to see whether they match the 7 Hz oscillations observed in the Busch and VanRullen study. On the other hand, the analysis of our small set of recordings from neuronal pairs suggests that neurons that share sensory preferences (with respect to motion direction in our case) tend to encode the same of two stimuli at a given time while neurons with different preferences tend to anti-correlate in their response states. This supports the hypothesis that the whole population of neurons responding to a given stimulus configuration tends to alternate their individual response states in a coordinated fashion.

The serial multiplexing we observe also allows us to account for other observations when multiple stimuli are combined within the same receptive field. This is most apparent for the case in which two RDPs moving in different directions are spatially superimposed, creating the percept of two surfaces sliding across each other. As documented in Treue et al. (2000), combining two directions with an angular separation of 30–60° creates a stimulus in which the two component motions are easily distinguishable perceptually, but causes a neural population response (averaged across trials) that is single-lobed, suggestive of a single direction in the receptive field. While the perception of two directions under such conditions can be explained by assuming a particular decoding mechanism, our observed multiplexing of the individual stimulus representations provides other types of explanation for the apparent discrepancy between neural responses and perception. Additionally, the distinct encoding of the two motion surfaces through separate response states might also allow the visual system to separately manipulate the individual stimulus representations as apparent in the perceptual (Marshak and Sekuler, 1979) and physiological (Helmer et al., 2016) repulsion of the perceived angular separation in such transparent motion patterns.

In summary, this study suggests and documents a neuronal coding scheme that temporally multiplexes information from multiple stimuli within the receptive fields of neurons in extrastriate visual cortex. This allows nervous systems to enjoy the benefits of large receptive fields (spatial integration of information to achieve more complex selectivities) without suffering from the disadvantage that large receptive fields pool the responses to multiple stimuli and thus lose critical information about their individual contribution to the cell's overall response. Such a system could also reconcile the observation of perceptual separability of multiple stimuli (such as surfaces in transparent motion) with the apparent pooling of information within the spatial extent of receptive fields in extrastriate visual cortex.

## Author contributions

KL, SK, SD, and CB conceptualized the research. VK and ST designed and performed experiments. KL and SD designed the statistical methodology. KL performed the analysis and prepared figures. All authors interpreted the results. KL, ST, SD, and CB wrote the paper. All authors approved the final version of the paper.

## Funding

The work was part of the Dynamical Systems Interdisciplinary Network, University of Copenhagen. The work of VK and ST was supported by the Volkswagen Foundation (grant I/79868), the Bernstein Center of Computational Neuroscience Göttingen (grants 01GQ0433 and 01GQ1005C) of the BMBF and the German Research Foundation (DFG) Research Unit 1847 “The Physiology of Distributed Computing Underlying Higher Brain Functions in Non-Human Primates”.

### Conflict of interest statement

The authors declare that the research was conducted in the absence of any commercial or financial relationships that could be construed as a potential conflict of interest.

## References

[B1] Britten K. H. Heuer H. W. (1999). Spatial summation in the receptive fields of MT neurons. J. Neurosc. 19, 5074–5084. 10366640 10.1523/JNEUROSCI.19-12-05074.1999 PMC6782635

[B2] Brown E. N. Barbieri R. Ventura V. Kass R. E. Frank L. M. (2002). The time-rescaling theorem and its application to neural spike train data analysis. Neural Comput. 14, 325–346. 10.1162/08997660252741149 11802915

[B3] Bundesen C. (1990). A theory of visual attention. Psychol. Rev. 97:523. 2247540 10.1037/0033-295x.97.4.523

[B4] Bundesen C. Habekost T. (2008). Principles of Visual Attention: Linking Mind and Brain. Oxford: Oxford University Press.

[B5] Bundesen C. Habekost T. Kyllingsbæk S. (2005). A neural theory of visual attention: bridging cognition and neurophysiology. Psychol. Rev. 112:291. 10.1037/0033-295X.112.2.291 15783288

[B6] Burnham K. P. Anderson D. R. (2002). Model Selection and Multimodel Inference: A Practical Information-Theoretic Approach. New York, NY: Springer.

[B7] Busch N. A. VanRullen R. (2010). Spontaneous eeg oscillations reveal periodic sampling of visual attention. Proc. Natl. Acad. Sci. U.S.A. 107, 16048–16053. 10.1073/pnas.1004801107 20805482 PMC2941320

[B8] Busse L. Wade A. R. Carandini M. (2009). Representation of concurrent stimuli by population activity in visual cortex. Neuron 64, 931–942. 10.1016/j.neuron.2009.11.004 20064398 PMC2807406

[B9] Calapai A. Berger M. Niessing M. Heisig K. Brockhausen R. Treue S.. (2016). A cage-based training, cognitive testing and enrichment system optimized for rhesus macaques in neuroscience research. Behav. Res. Methods. 10.3758/s13428-016-0707-3. [Epub ahead of print]. Available online at: http://link.springer.com/article/10.3758/s13428-016-0707-3 26896242 PMC5352800

[B10] Claeskens G. Hjort N. L. (2008). Model Selection and Model Averaging, Vol. 330. Cambridge: Cambridge University Press.

[B11] Cox D. R. Lewis P. A. (1966). The Statistical Analysis of Series of Events. London: Chapman and Hall.

[B12] Cui Y. Liu L. D. McFarland J. M. Pack C. C. Butts D. A. (2016). Inferring cortical variability from local field potentials. J. Neurosci. 36, 4121–4135. 10.1523/JNEUROSCI.2502-15.2016 27053217 PMC4821918

[B13] Daley D. J. Vere-Jones D. (1988). An Introduction to the Theory of Point Processes, Vol. 2. New York, NY: Springer.

[B14] Desimone R. Duncan J. (1995). Neural mechanisms of selective visual attention. Annu. Rev. Neurosci. 18, 193–222. 7605061 10.1146/annurev.ne.18.030195.001205

[B15] Fiebelkorn I. C. Saalmann Y. B. Kastner S. (2013). Rhythmic sampling within and between objects despite sustained attention at a cued location. Curr. Biol. 23, 2553–2558. 10.1016/j.cub.2013.10.063 24316204 PMC3870032

[B16] Gattass R. Nascimento-Silva S. Soares J. G. Lima B. Jansen A. K. Diogo A. C.. (2005). Cortical visual areas in monkeys: location, topography, connections, columns, plasticity and cortical dynamics. Philos. Transact. R. Soc. Lond. B 360, 709–731. 10.1098/rstb.2005.1629 15937009 PMC1569490

[B17] Gilmore R. O. Hou C. Pettet M. W. Norcia A. M. (2007). Development of cortical responses to optic flow. Vis. Neurosci. 24, 845–856. 10.1017/S0952523807070769 18093371

[B18] Goris R. L. Movshon J. A. Simoncelli E. P. (2014). Partitioning neuronal variability. Nat. Neurosci. 17, 858–865. 10.1038/nn.3711 24777419 PMC4135707

[B19] Harrell F. E. (2001). Regression Modeling Strategies. New York, NY: Springer Science & Business Media.

[B20] Hartigan J. A. Hartigan P. M. (1985). The dip test of unimodality. Ann. Stat. 13, 70–84.

[B21] Haslinger R. Pipa G. Brown E. (2010). Discrete time rescaling theorem: determining goodness of fit for discrete time statistical models of neural spiking. Neural Comput. 22, 2477–2506. 10.1162/NECO_a_00015 20608868 PMC2932849

[B22] Helmer M. Kozyrev V. Stephan V. Treue S. Geisel T. Battaglia D. (2016). Model-free estimation of tuning curves and their attentional modulation, based on sparse and noisy data. PLoS ONE 11:e146500. 10.1371/journal.pone.0146500 26785378 PMC4718600

[B23] Jones D. R. Perttunen C. D. Stuckman B. E. (1993). Lipschitzian optimization without the lipschitz constant. J. Optim. Theory Appl. 79, 157–181. 10.1007/BF00941892

[B24] Kanwisher N. Yovel G. (2006). The fusiform face area: a cortical region specialized for the perception of faces. Philos. Trans. R. Soc. Lond. B 361, 2109–2128. 10.1098/rstb.2006.1934 17118927 PMC1857737

[B25] Kass R. E. Eden U. T. Brown E. N. (2014). Analysis of Neural Data. New York, NY: Springer.

[B26] Katzner S. Busse L. Treue S. (2009). Attention to the color of a moving stimulus modulates motion-signal processing in macaque area mt: evidence for a unified attentional system. Front. Syst. Neurosci. 3:12. 10.3389/neuro.06.012.2009 19893762 PMC2773174

[B27] Latimer K. W. Yates J. L. Meister M. L. Huk A. C. Pillow J. W. (2015). Single-trial spike trains in parietal cortex reveal discrete steps during decision-making. Science 349, 184–187. 10.1126/science.aaa4056 26160947 PMC4799998

[B28] Lee J. Maunsell J. H. (2009). A normalization model of attentional regulation of single unit responses. PLoS ONE 4:e4651. 10.1371/journal.pone.0004651 19247494 PMC2645695

[B29] Li K. Vozyrev V. Kyllingsbæk S. Treue S. Ditlevsen S. Bundesen C. (2016). Data from: Neurons in primate visual cortex alternate between responses to multiple stimuli in their receptive field. Dryad Digit. Repos. 10.5061/dryad.88pv1 PMC5187355 28082892

[B30] MacEvoy S. P. Tucker T. R. Fitzpatrick D. (2009). A precise form of divisive suppression supports population coding in the primary visual cortex. Nat. Neurosci. 12, 637–645. 10.1038/nn.2310 19396165 PMC2875123

[B31] Majaj N. J. Carandini M. Movshon J. A. (2007). Motion integration by neurons in macaque mt is local, not global. J. Neurosci. 27, 366–370. 10.1523/JNEUROSCI.3183-06.2007 17215397 PMC3039841

[B32] Marshak W. Sekuler R. (1979). Mutual repulsion between moving visual targets. Science 205, 1399–1401. 472756 10.1126/science.472756

[B33] Martinez-Trujillo J. Treue S. (2004). Feature-based attention increases the selectivity of population responses in primate visual cortex. Curr. Biol. 14, 744–751. 10.1016/j.cub.2004.04.028 15120065

[B34] Martınez-Trujillo J. C. Treue S. (2002). Attentional modulation strength in cortical area mt depends on stimulus contrast. Neuron 35, 365–370. 10.1016/S0896-6273(02)00778-X 12160753

[B35] Nandy A. S. Sharpee T. O. Reynolds J. H. Mitchell J. F. (2013). The fine structure of shape tuning in area V4. Neuron 78, 1102–1115. 10.1016/j.neuron.2013.04.016 23791199 PMC3694358

[B36] Nelder J. A. Mead R. (1965). A simplex method for function minimization. Comput. J. 7, 308–313.

[B37] Ni A. M. Ray S. Maunsell J. H. (2012). Tuned normalization explains the size of attention modulations. Neuron 73, 803–813. 10.1016/j.neuron.2012.01.006 22365552 PMC3292773

[B38] Niebergall R. Khayat P. S. Treue S. Martinez-Trujillo J. C. (2011a). Expansion of mt neurons excitatory receptive fields during covert attentive tracking. J. Neurosci. 31, 15499–15510. 10.1523/JNEUROSCI.2822-11.2011 22031896 PMC6703514

[B39] Niebergall R. Khayat P. S. Treue S. Martinez-Trujillo J. C. (2011b). Multifocal attention filters targets from distracters within and beyond primate mt neurons' receptive field boundaries. Neuron 72, 1067–1079. 10.1016/j.neuron.2011.10.013 22196340

[B40] Orhan A. E. Ma W. J. (2015). Neural population coding of multiple stimuli. J. Neurosci. 35, 3825–3841. 10.1523/JNEUROSCI.4097-14.2015 25740513 PMC4461696

[B41] Patzwahl D. R. Treue S. (2009). Combining spatial and feature-based attention within the receptive field of mt neurons. Vis. Res. 49, 1188–1193. 10.1016/j.visres.2009.04.003 19362573

[B42] Press W. H. (2007). Numerical Recipes: The Art of Scientific Computing, 3rd Edn. Cambridge: Cambridge University Press.

[B43] Recanzone G. H. Wurtz R. H. Schwarz U. (1997). Responses of MT and MST neurons to one and two moving objects in the receptive field. J. Neurophysiol. 78, 2904–2915. 9405511 10.1152/jn.1997.78.6.2904

[B44] Reynolds J. H. Chelazzi L. Desimone R. (1999). Competitive mechanisms subserve attention in macaque areas V2 and V4. J. Neurosci. 19, 1736–1753. 10024360 10.1523/JNEUROSCI.19-05-01736.1999 PMC6782185

[B45] Reynolds J. H. Heeger D. J. (2009). The normalization model of attention. Neuron 61, 168–185. 10.1016/j.neuron.2009.01.002 19186161 PMC2752446

[B46] Reynolds J. H. Pasternak T. Desimone R. (2000). Attention increases sensitivity of v4 neurons. Neuron 26, 703–714. 10.1016/S0896-6273(00)81206-4 10896165

[B47] Seidemann E. Newsome W. T. (1999). Effect of spatial attention on the responses of area mt neurons. J. Neurophysiol. 81, 1783–1794. 10200212 10.1152/jn.1999.81.4.1783

[B48] Shokhirev K. Kumar T. Glaser D. (2006). The influence of cortical feature maps on the encoding of the orientation of a short line. J. Comput. Neurosci. 20, 285–297. 10.1007/s10827-006-6485-7 16683208

[B49] Smith A. T. Singh K. D. Williams A. L. Greenlee M. W. (2001). Estimating receptive field size from fMRI data in human striate and extrastriate visual cortex. Cereb. Cortex 11, 1182–1190. 10.1093/cercor/11.12.1182 11709489

[B50] Snowden R. J. Treue S. Erickson R. G. Andersen R. A. (1991). The response of area mt and v1 neurons to transparent motion. J. Neurosci. 11, 2768–2785. 1880548 10.1523/JNEUROSCI.11-09-02768.1991 PMC6575251

[B51] Treue S. Hol K. Rauber H. J. (2000). Seeing multiple directions of motion-physiology and psychophysics. Nat. Neurosci. 3, 270–276. 10.1038/72985 10700260

[B52] Treue S. Trujillo J. (1999). Feature-based attention influences motion processing gain in macaque visual cortex. Nature 399, 575–579. 10376597 10.1038/21176

[B53] Treue S. Martínez Trujillo J. C.. (1999). Feature-based attention influences motion processing gain in macaque visual cortex. Nature 399, 575–579. 10376597 10.1038/21176

[B54] Truccolo W. Eden U. T. Fellows M. R. Donoghue J. P. Brown E. N.. (2005). A point process framework for relating neural spiking activity to spiking history, neural ensemble, and extrinsic covariate effects. J. Neurophysiol. 93, 1074–1089. 10.1152/jn.00697.2004 15356183

[B55] Xue C. Kaping D. Baloni R. S. Krishna B. S. Treue S. (2016). Spatial attention reduces burstiness in macaque visual cortical area MST. Cereb. Cortex. 10.1093/cercor/bhw326 [Epub ahead of print]. Available online at: http://cercor.oxfordjournals.org/content/early/2016/11/22/cercor.bhw326.abstract PMC5939203 28365773

[B56] Zoccolan D. Cox D. D. DiCarlo J. J. (2005). Multiple object response normalization in monkey inferotemporal cortex. J. Neurosci. 25, 8150–8164. 10.1523/JNEUROSCI.2058-05.2005 16148223 PMC6725538

